# Facile synthesis of triazolo/benzazolo[2,1-*b*]quinazolinone derivatives catalyzed by a new deep eutectic mixture based on glucose, pregabalin and urea†

**DOI:** 10.1039/d3ra05199d

**Published:** 2023-10-27

**Authors:** Parissa Naddaf Rahro, Farhad Shirini, Ali Ghanadzadeh Gilani

**Affiliations:** a Department of Chemistry, College of Sciences, University of Guilan Rasht 41335-19141 Iran shirini@guilan.ac.ir fshirini@gmail.com +98 131 3233262 +981313233262

## Abstract

In this study, a novel natural deep eutectic solvent was prepared from glucose, pregabalin, and urea. The prepared solvent was identified using a variety of techniques, including Fourier transform infrared spectroscopy (FT-IR), thermogravimetric analysis (TGA), derivative thermogravimetry (DTG), differential thermal analysis (DTA), and refractive index measurements (RI). The prepared deep eutectic solvent was then utilized for the one-pot synthesis of quinazolinone derivatives. The yields of the product obtained with and without the catalyst were determined, providing insights into the catalytic efficiency of the system. This protocol offers several advantages, including mild reaction conditions, easy reagent preparation, a green process, short reaction times (2–60 min), high yields (80–99%), and a straightforward procedure with the possibility of catalyst reusability.

## Abstract 2

In this study, a novel natural deep eutectic solvent was prepared from glucose, pregabalin, and urea.

## Graphical abstract

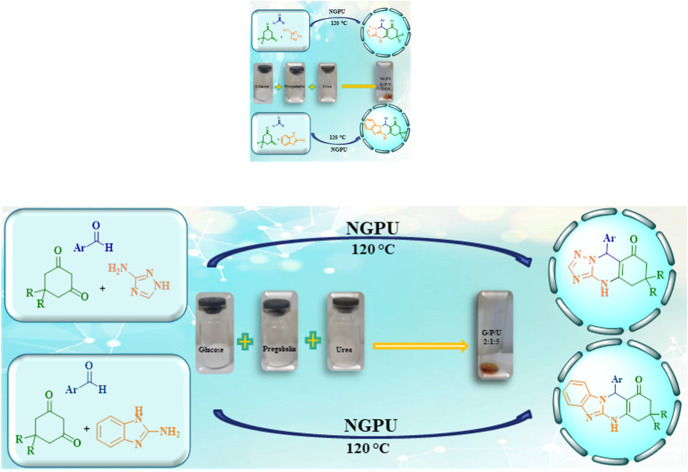

## Introduction

Easily vaporable mixtures with ecological benefits are rapidly gaining importance in modern chemistry because their use can reduce the environmental impact of organic solvents. Ionic liquids (ILs) play an important role in this area as a suitable green media and/or a promotor in organic synthesis. In these salts, poor ionic coordination and low salt melting points result from charge dispersion and/or delocalization (below 100 °C).^1–4^ ILs have many environmentally friendly properties, such as low vapor pressure, considerable stability under different thermal and chemical conditions ease of handling, high recyclability and low flammability.^1^ Therefore, ILs can play an important role as reaction and extraction media. Additionally, in some chemical reactions, the use of these liquids has enabled the production of products that are not obtainable using common organic solvents or simple post-treatment methods.^5–7^ Unfortunately, most of these compounds were found to be toxic, poorly biodegradable, and therefore poorly biocompatible and also showed some ecological disadvantages.^8,9^

Deep eutectic solvents (DES) are a new class of organic solvents which can be easily prepared by the mixing and heating of appropriate hydrogen bond donor (HBD) and hydrogen bond acceptor (HBA) molecules in the correct molar ratios. These hydrogen-bonding interactions cause the melting point of these mixtures be very low even at room temperature.^1,10^ It should be mentioned that because of the presence of salts in many of these systems, sometimes, they are classified as a subclass of ionic liquids.

One of the interesting features of these mixtures is their simple preparation method which can be done by mixing and heating two or more species of HBD and HBA molecules until a homogeneous liquid is formed. This process is cheap and “green” in terms of the atom economy of the process.^10^ A variety of starting materials can be used for the preparation of DESs and natural deep eutectic solvents (NADES),^11^ as a new class of these mixtures, of which metabolites,^12^ organic acids,^13,14^ amino acids,^15,16^ sugars,^17,18^ choline^19–21^ and urea^20,22^ are the most important ones.

In recent years, the synthesis of biologically active compounds *via* green and effective methods has become an attractive topic in organic chemistry.^23^ Among a variety of methods multi-component reactions (MCR) are one of the most important candidates for this purpose. This is because the principles, such as atom economy, effectiveness of the reactions and simplicity of processes from doing the reactions to the separation of the products, are well respected in these methods.^24,25^

Quinazolinone derivatives are an interesting type of heterocyclic compound showing considerable biological and pharmacological activities, including: Antivirals and antihistamines,^26^ analgesic and anti-inflammatory,^27^ antitumor,^28^ anticancer^29^ and anti-HIV^30^ activities. Use as potent immunosuppressants^28^ is another application of these compounds.

Triazolo[2,1-*b*]quinazolinones and benzazolo[2,1-*b*]quinazolinones are important derivatives of quinazolines. A common way for the preparation of these important target molecules is the multi-component reaction of aldehydes with 3-amino-1,2,4-triazole or 2-aminobenzimidazole, and a β-diketone in the presence of a variety of catalysts.^31–36^

Although the use of these catalysts causes an improvement, harsh reaction conditions, long reaction times, expensive reagents, low yields of the products and the use of a large quantity of volatile organic solvents are important limitations associated with these methods. Furthermore, during most of the existing methods, the catalyst cannot be recovered or reused. Therefore, the introduction of simple, efficient and mild procedures for the synthesis of the above-mentioned derivatives of quinazolinone is still needed. Also, other studies have reported the synthesis of quinazolinone derivatives using deep eutectic solvents.^37,38^ Referencing these studies demonstrates that the synthesis of quinazolinone derivatives in deep eutectic solvents has been explored in the literature. This highlights the significance of the current study and its contribution to the existing knowledge in the field. Herein, we wish to introduce a new natural deep eutectic mixture which can speed-up the mentioned reactions by removing some of the aforementioned limitations.

## Results and discussion

In the first stage of this study, various amounts of glucose, pregabalin, and urea were tested to prepare the desired deep eutectic mixture. The results indicated that the optimal results were achieved when these compounds were mixed in molar ratios of 1 : 1 : 5, 5 : 1 : 2, and 5 : 1 : 3, and heated at 110 °C for 30 minutes (Table 1, entries 6, 8 and 10).

**Table 1:** Preparation of deep eutectic mixture

Entry	Mixture	Molar ratio	Appearance at room temperature
1	Glucose/pregabalin/urea	1 : 1 : 1	Solid
2	Glucose/pregabalin/urea	1 : 1 : 2	Solid
3	Glucose/pregabalin/urea	1 : 1 : 3	Solid
4	Glucose/pregabalin/urea	1 : 1 : 4	Solid
5	Glucose/pregabalin/urea	2 : 1 : 4	Solid
6	Glucose/pregabalin/urea	1 : 1 : 5	Clear liquid
7	Glucose/pregabalin/urea	1 : 1 : 6	Sticky paste
8	Glucose/pregabalin/urea (NGPU)	2 : 1 : 5	Clear liquid
9	Glucose/pregabalin/urea	2 : 1 : 4	Solid
10	Glucose/pregabalin/urea	3 : 1 : 5	Clear liquid
11	Glucose/pregabalin/urea	1 : 2 : 5	Solid
12	Glucose/pregabalin/urea	1 : 3 : 5	Solid

To determine the best ratio in terms of catalytic properties, the catalytic properties of the deep eutectic mixtures were tested in the synthesis of triazolo/benzazolo[2,1-*b*]quinazolinone, as shown in Tables 2 and 4. The deep eutectic mixture with a molar ratio of 2 : 1 : 5 (referred to as NGPU in this article) demonstrated the best result (Tables 2, entry 3 and 4, entry 4).

**Table 2:** Optimization of the reaction conditions for the synthesis of the triazolo[2,1-*b*]quinazolinone derivative of 4-chlorobenzaldehyde catalyzed by NGPU

Entry	Catalyst (mol%)	Solvent	Temperature (°C)	Time (min)	Conversion (%)
1	NGPU (2)	—	120	40	100
2	NGPU (3)	—	120	40	Mixture of products
**3**	**NGPU (4)**	**—**	**120**	**20**	**100**
4	NGPU (8)	—	120	43	100
5	NGPU (8)	—	100	40	Not completed
6	NGPU (4)	—	70	56	Not completed
7	NGPU (4)	EtOH	Reflux	50	Not completed
8	NGPU (4)	CH_3_CN	Reflux	50	Not completed
9	NGPU (4)	H_2_O	Reflux	50	Not completed
10	Glucose/pregabalin/urea (4) 1 : 1 : 5	—	120	35	100
11	Glucose/pregabalin/urea (4) 3 : 1 : 5	—	120	40	100
12	Pregabalin (4)	—	120	100	Not completed
13	Glucose (4)	—	120	100	Not completed
14	Urea (4)	—	120	100	Mixture of products
15	—	—	120	100	Trace

After preparation, the selected deep eutectic mixture (NGPU) was identified using methods commonly used to identify this group of mixtures. The results of the identification are described in the next section.

### Characterization of the catalyst

#### FT-IR analysis

**Fig. 1:**
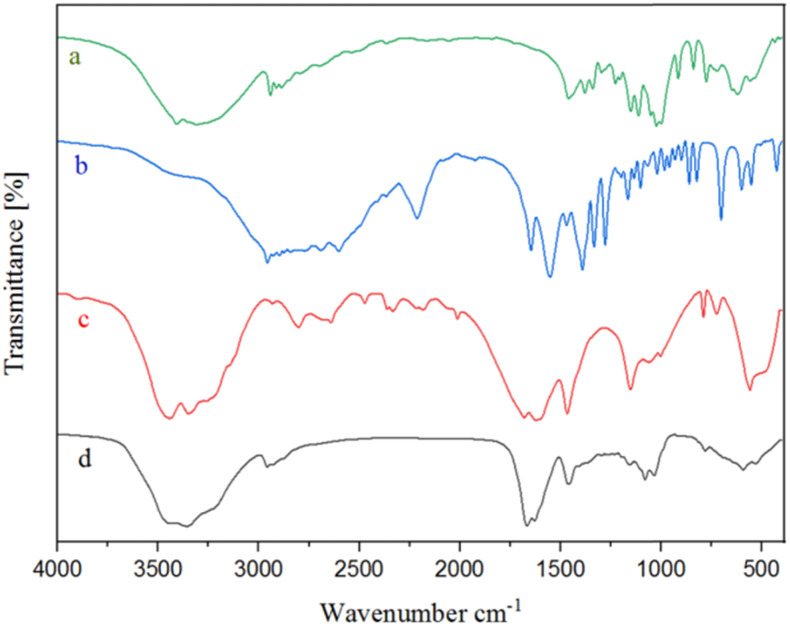
FT-IR spectra of glucose (a), pregabalin (b), urea (c) and the prepared NGPU (d).

The FT-IR spectra of NGPU, glucose, pregabalin, and urea were compared and are presented in Fig. 1. The comparison showed that the FT-IR spectrum of NGPU tends to resemble each of its constituent pure components. For instance, in the FT-IR spectrum of the prepared NGPU, the peaks in the 3227–3445 cm^−1^ region are related to the vibrations of –OH and –NH in glucose, pregabalin and urea. Also, the average peak observed at 2957 cm^−1^ is related to the vibrations of the aliphatic CH bonds. In this context the sharp peaks at 1628 and 1665 cm^−1^ correspond to the N–H and C=O tensile vibrations, respectively. Furthermore, the absorption peaks around 1034 and 1079 cm^−1^ are related to the C–N bending vibrations of pregabalin and urea.

Furthermore, shifts of certain peaks and weakening of the vibrations of several functional groups were also observed and these changes were attributed to the formation of hydrogen bonds between the components of NGPU and the resulting formation of NGPU.

#### TGA analysis

**Fig. 2:**
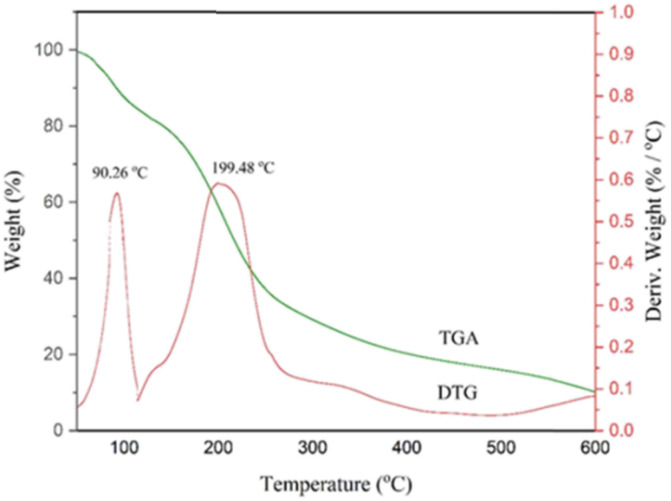
TGA–DTG curve of NGPU.

**Fig. 3:**
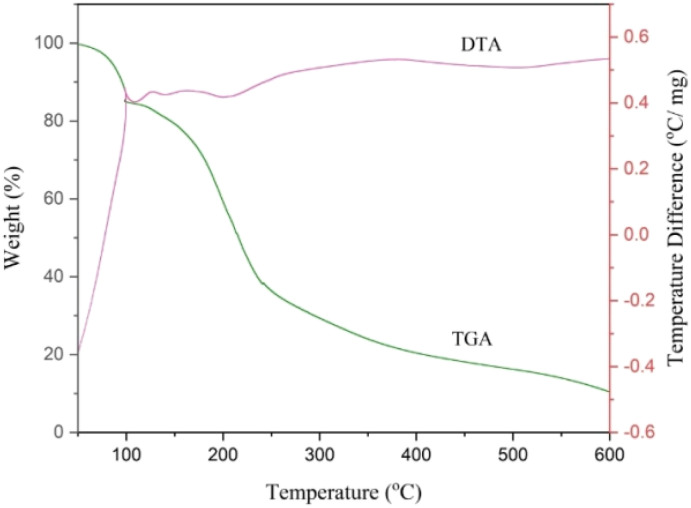
TGA–DTA curve of NGPU.

Thermogravimetric analysis (TGA) was used for the determination of the thermal behavior of NGPU. The range of this analysis was selected from room temperature to 600 °C (Fig. 2 and 3). The TGA curve shows a weight loss below 100 °C, which can be due to the loss of physically absorbed water (7.11%). It also consists of only one weight loss step with a maximum DTG of 199.48 °C in the range of 120–250 °C, attributable to the decomposition of the organic parts, while pure glucose at 176.8 °C,^39^ pregabalin at 205 °C (ref. 40) and urea up to 215 °C (ref. 41) are decomposed. This difference could be due to the interaction between the components of DES, which leads to the formation of a single pseudo-molecule.

#### Refractive index measurement

The refractive index value of the prepared NGPU was studied at 298 K. Based on multiple measurements (at least three), the refractive index measurement uncertainty was estimated to be 0.0005 *n*_D_. At this temperature, a refractive index value of 1.4935 *n*_D_ was recorded.

#### Measurement of absorption spectra and polarity

The polarity of NGPU was characterized by a well-known solvent polarity parameter or dielectric constant (*ε*) through solvatochromic analysis.

**Fig. 4:**
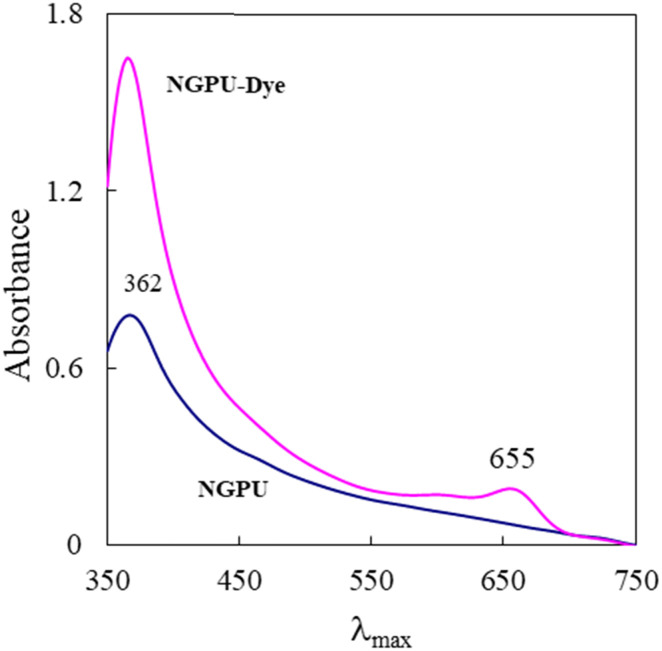
Visible absorption spectra of NGPU with and without the oxazine dye.

Fig. 4 shows the visible absorption spectrum of oxazine 1 perchlorate (OX1) in a prepared deep eutectic solvent. The absorption spectrum of the pure NGPU is overplotted for comparison purposes. The spectrum of the dye typically possesses an intense band, which is neighbored by a shoulder at shorter wavelengths.

**Fig. 5:**
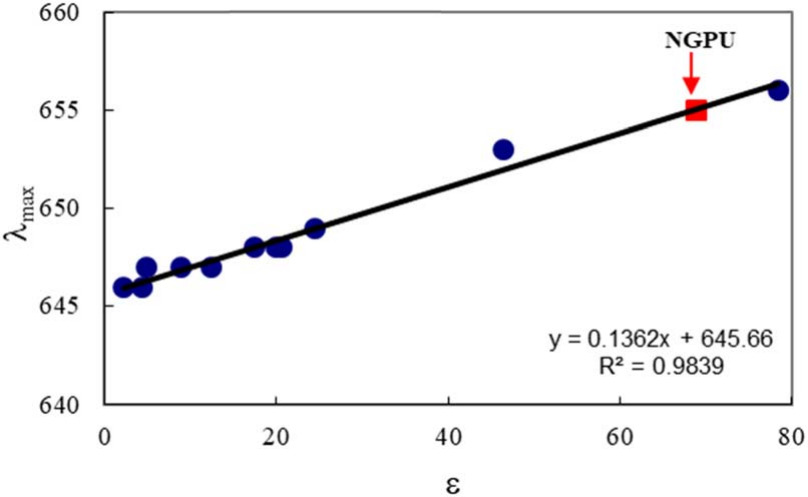
Variation of absorption maximum wavelength (*λ*_max_) of OX1 dye (2 × 10^−5^ M) as a function of the solvent dielectric constant, *ε*, at room temperature; the selected solvents were mainly low polarity and polar protic solvents, *i.e.* 1,4-dioxane, diethyl ether, chloroform, dichloromethane, *t*-butanol, 1-butanol, 2-propanol, acetone, ethanol, water (the dielectric data are taken from ref. 42).

Fig. 5 shows the variation in maximum absorption wavelength of oxazine 1 perchlorate (a solvatochromic dye) as a function of solvent polarity (dielectric constant). As can be seen, a relatively good correlation was observed between *λ*_max_ values and the selected solvent, and a regression of 0.9925 was obtained for this scale. The dye exhibited an absorption maximum at 655 nm in the DES. According to the polarity curve, the prepared DES shows a high polarity and its dielectric constant was estimated to be about *ε* = 71.5.

The absorption spectrum of oxazine 1 in NGPU is red shifted as compared to the dye spectra in normal organic solvents (non-polar and low polarity media). The red shift observed for the dye in the prepared DES indicates strong intermolecular interactions between the dye molecules and the polar DES medium. Although based on the previous report,^43^ oxazine-1 was considered as a poor polarity indicator. However, the spectral profile shows a regular variation on going from the non-polar or low polarity solvents to the polar protic solvents.

### Catalytic activity

**Scheme 1:**
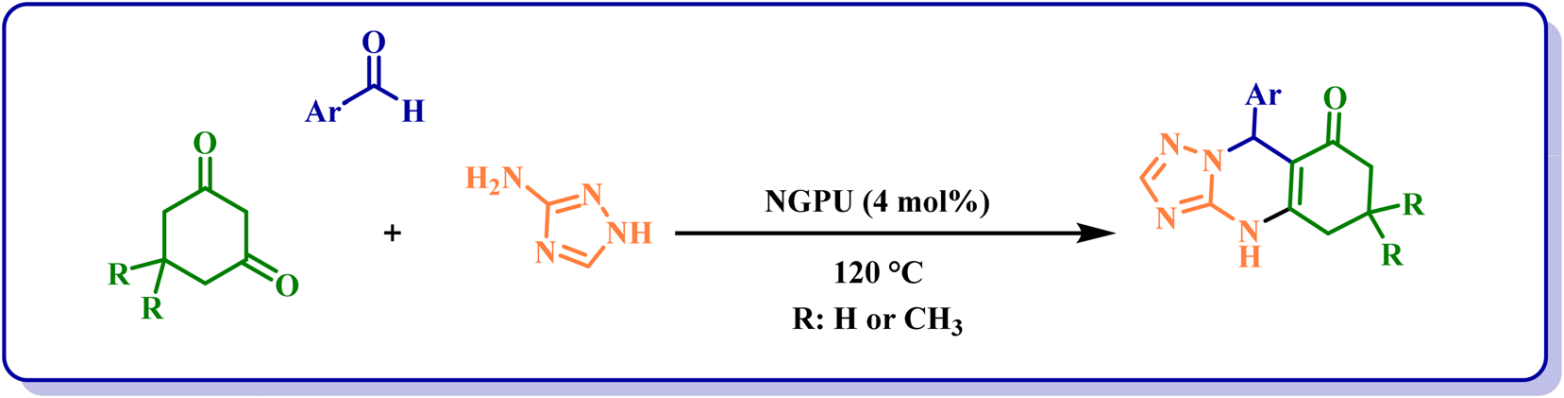
Synthesis of triazolo[2,1-*b*]quinazolinone derivatives catalyzed by NGPU.

After the identification of the produced NGPU, its activity in the promotion of the synthesis of triazolo[2,1-*b*]quinazolinones and benzazolo[2,1-*b*] derivatives was investigated. In the first step, the one pot three-component reaction of 4-chlorobenzaldehyde, dimedone and 3-amino-1,2,4-triazole was selected as a model to study the influence of different conditions on it and the obtained results (Table 2) clarified that the suitable conditions for obtaining the best results is as shown in Scheme 1. Based on the obtained data, we should pay our attention to the following points: (a) the reaction cannot proceed in the presence of starting materials used for the preparation of NGPU (entries 12–14); (b) the reaction in the solvent or at lower temperatures remains incomplete even after prolonged heating (entries 5–9) and (c) a variety of molar ratios of glucose, pregabalin and urea were used for the preparation of NGPU and the one with the molar ratio of 2 : 1 : 5 led to the best results in 4 mol%. It should be mentioned that, because we used catalytic amounts of the prepared reagent, in this study the term deep eutectic mixture (DEM) is used instead of deep eutectic solvent (DES).

Based on the results obtained from the above-mentioned preliminary studies, the efficiency of this protocol was studied for the reaction of a variety of aromatic aldehydes containing different types of substituents (Table 3). Using this method, high yields of the desired products were isolated in short reaction times.

**Table 3:** Synthesis of triazolo[2,1-*b*]quinazolinone derivatives catalyzed by NGPU^a^

Entry	Ar	R	Product	Time (min)	Yield^b^ (%)	M.p. (°C)	
						Found	Reported
1	C_6_H_5_	CH_3_	1a	22	96	254–257	252–254 (ref. 26)
2	4-Cl-C_6_H_4_	CH_3_	2a	20	94	300–303	304–305 (ref. 44)
3	2-Cl-C_6_H_4_^c^	CH_3_	3a	14	88	286–290	288–290 (ref. 45)
4	2,4-Cl_2_-C_6_H_3_	CH_3_	4a	20	90	>300	>300 (ref. 46)
5	4-Br-C_6_H_4_	CH_3_	5a	30	85^d^	292–295	287–288 (ref. 44)
6	4-NO_2_-C_6_H_4_	CH_3_	6a	20	91	291–293	290–294 (ref. 47)
7	3-NO_2_-C_6_H_4_	CH_3_	7a	22	93	292–294	290–293 (ref. 46)
8	4-OCH_3_-C_6_H_4_	CH_3_	8a	20	95	233–235	231–233 (ref. 46)
9	4-OH-C_6_H_4_	CH_3_	9a	18	97	>300	>300 (ref. 31)
10	4-OH-3-OCH_3_-C_6_H_3_	CH_3_	10a	25	85^d^	272–277	287–290 (ref. 48)
11	4-CH_3_-C_6_H_4_	CH_3_	11a	20	93	262–266	265–267 (ref. 45)
12	2-CH_3_-C_6_H_4_	CH_3_	12a	16	97	305–306	299–300 (ref. 28)
13	C_6_H_5_	H	13a	24	98	298–299	296–300 (ref. 32)
14	4-Cl-C_6_H_4_	H	14a	60	80^d^	294–295	294–296 (ref. 32)
15	3,4,5-(OCH_3_)_3_–C_6_H_2_	H	15a	4	98	>300	297–299 (ref. 49)
16	4-Br-C_6_H_4_	H	16a	27	98	>300	306–308 (ref. 32)
17	4-CH_3_-C_6_H_4_	H	17a	7	97	>300	316–318 (ref. 50)
18	3-NO_2_-C_6_H_4_	H	18a	16	90	298–300	292–296 (ref. 32)
19	2-CH_3_-C_6_H_4_	H	19a	5	98	>300	New
20	2-Br-C_6_H_4_	H	20a	5	97	>300	New
21	4-SCH_3_-C_6_H_4_	H	21a	3	95	>300	New

a Reaction conditions: aldehyde (1 mmol), 1,3-diketones (dimedone and/or 1,3-cyclohexanediones) (1 mmol) and 3-amino-1,2,4-triazole (1 mmol).

b Isolated yields.

c 8 mol% NGPU (2 : 1 : 5).

d Isolated yields after recrystallization.

**Scheme 2:**
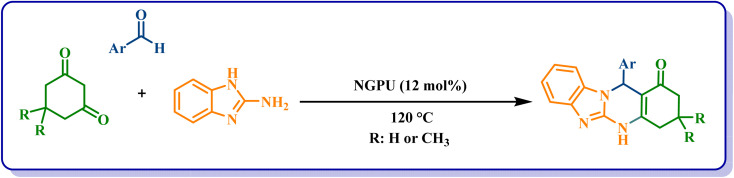
Synthesis of benzazolo[2,1-*b*]quinazolinone derivatives catalyzed by NGPU.

In the next step, the effectiveness of NGPU as the catalyst was studied in the preparation of benzazolo[2,1-*b*]quinazolinones (Scheme 2). Firstly, the reaction of 4-chlorobenzaldehyde, dimedone and 2-aminobenzimidazole was studied under the influence of different factors and the best condition was selected according to entry 4 from Table 4. Then the reaction was carried out on aromatic aldehydes containing both electron-withdrawing and electron-donating substituents and all of the named target compounds were obtained with high yields in short reaction times (Table 5).

**Table 4:** Optimization of the reaction conditions for the synthesis of the benzazolo[2,1-*b*]quinazolinone derivative of 4-chlorobenzaldehyde catalyzed by NGPU

Entry	Catalyst (mol%)	Solvent	Temperature (°C)	Time (min)	Conversion (%)
1	NGPU (2)	—	120	90	100
2	NGPU (4)	—	120	40	100
3	NGPU (8)	—	120	25	100
**4**	**NGPU (12)**	**—**	**120**	**22**	**100**
5	NGPU (16)	—	120	28	100
6	NGPU (12)	—	100	45	100
7	NGPU (12)	EtOH	Reflux	80	Not completed
8	NGPU (12)	CH_3_CN	Reflux	80	Not completed
9	NGPU (12)	H_2_O	Reflux	80	Mixture of products
10	Glucose/pregabalin/urea (12) 1 : 1 : 5	—	120	40	100
11	Glucose/pregabalin/urea (12) 3 : 1 : 5	—	120	45	100
12	Pregabalin (4)	—	120	100	Not completed
13	Glucose (4)	—	120	100	Not completed
14	Urea (4)	—	120	100	Mixture of products
15	—	—	120	100	Trace

**Table 5:** Synthesis of various benzazolo[2,1-*b*]quinazolinones catalyzed by NGPU.^a^

Entry	Ar	R	Product	Time (min)	Yield^b^ (%)	M.p. (°C)	
						Found	Reported
1	C_6_H_5_	CH_3_	1b	29	99	>300	>300 (ref. 28)
2	4-Cl–C_6_H_4_	CH_3_	2b	22	98	>300	>300 (ref. 32)
3	4-Br–C_6_H_4_	CH_3_	3b	33	98	>300	>300 (ref. 32)
4	4-OH–C_6_H_4_	CH_3_	4b	14	99	>300	>300 (ref. 52)
5	2,4-Cl_2_–C_6_H_3_	CH_3_	5b	27	98	>300	>300 (ref. 32)
6	3,4,5-(OCH_3_)_3_–C_6_H_2_	CH_3_	6b	12	95	>300	>300 (ref. 53)
7	4-CH_3_–C_6_H_4_	CH_3_	7b	13	99	>300	>300 (ref. 32)
8	2-CH_3_–C_6_H_4_	CH_3_	8b	8	99	>300	>300 (ref. 28)
9	3-NO_2_–C_6_H_4_	CH_3_	9b	18	85^c^	>300	>300 (ref. 32)
10	C_6_H_5_	H	10b	6	98	>300	310–312 (ref. 54)
11	2-CH_3_–C_6_H_4_	H	11b	15	96	>300	New
12	2-Br–C_6_H_4_	H	12b	7	89	>300	New
13	3,4,5-(OCH_3_)_3_–C_6_H_2_	H	13b	2	99	>300	>300 (ref. 55)
14	4-Pyridinebenzaldehyde	H	14b	18	95	>300	New
15	4-NO_2_–C_6_H_4_	H	15b	20	95	>300	>300 (ref. 54)
16	4-F-C_6_H_4_	H	16b	24	90	>300	>300 (ref. 54)

a Reaction conditions: aldehyde (1 mmol), 1,3-diketones (dimedone and/or 1,3-cyclohexanediones) (1 mmol), 2-aminobenzimidazole (1 mmol).

b Isolated yields.

c Isolated yields after recrystallization.

**Scheme 3:**
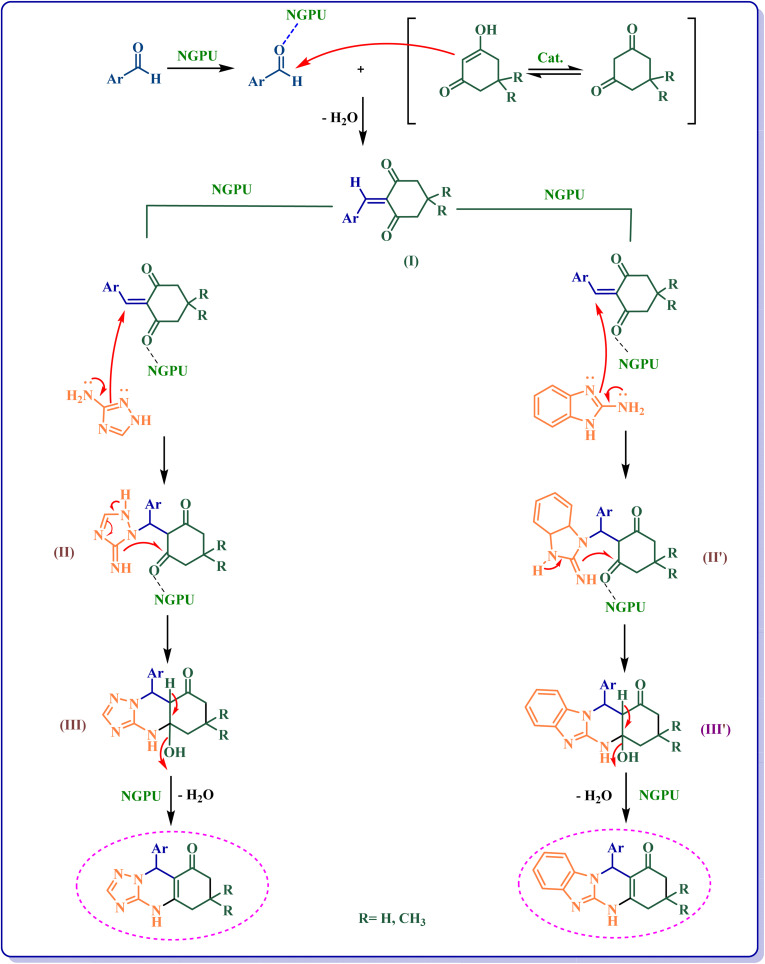
The proposed mechanism of the studied reactions.

Scheme 3 shows the probable pathway of the synthesis of [1,2,4]triazoloquinazolinones and benzimidazoquinazolinones in the presence of NGPU. According to this mechanism, NGPU can activate the aldehyde against the nucleophilic attack of β-diketone leading to intermediate I. The reaction of this intermediate and nitrogen number 2 of 3-amino-1,2,4-triazole or 2-amino-benzimidazole *via* Michael addition produces intermediate II or II′. Then these intermediates lead to III or III′ which, by intermolecular cyclization followed by removal of a molecule of water, afford the desired products.^51^

**Fig. 6:**
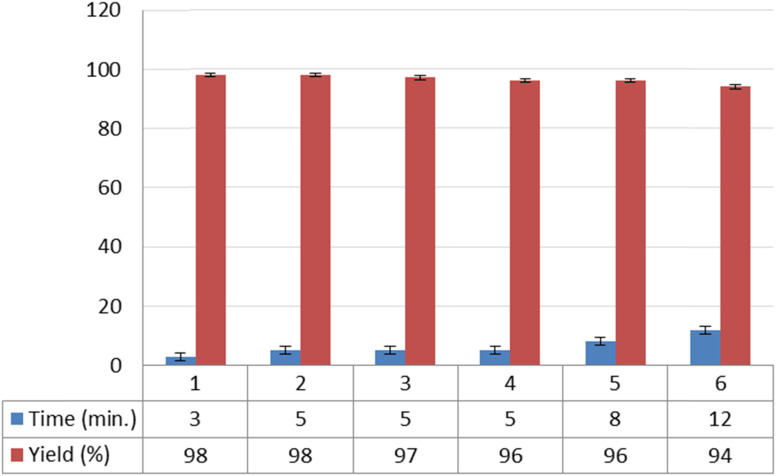
Reusability of the catalyst.

Reusability is a key feature of a catalyst and shows its compatibility with the rules of green chemistry. So to investigate this feature for NGPU, the synthesis of 9-(4-hydroxyphenyl)-6,6-dimethyl-5,6,7,9-tetrahydro-[1,2,4]triazolo [5,1-*b*]quinazolin-8(4*H*)-one was studied as the model one. At the end of the reactions, water was added and stirred for 10 min (NGPU is soluble in water), the mixture was filtered off and the solvent was evaporated from the filtrate under vacuum (70 °C). This procedure was repeated six times, and each time the desired product was obtained with an insignificant variation in reaction time and yield, a result which clarifies the practical recyclability of the catalyst (Fig. 6). The FT-IR of the same recovered and freshly prepared NGPU shows its stability under the selected conditions.

Table 6 compares the results obtained from the synthesis of 9-(4-hydroxyphenyl)-6,6-dimethyl-5,6,7,9-tetrahydro-[1,2,4] triazolo[5,1-*b*]quinazolin-8(4*H*)-one and 12-(4-hydroxyphenyl)-3,3-dimethyl-3,4,5,12-tetrahydrobenzo[4,5]imidazo[2,1-*b*] quinazolin-1(2*H*)-one in the presence of NGPU with some of the previously reported catalysts. This comparison is good evidence to accept that the present method is superior in terms of the efficiency, reaction times and the catalyst amounts.

**Table 6:** Comparison of the results obtained from the synthesis of 9-(4-hydroxyphenyl)-6,6-dimethyl-5,6,7,9-tetrahydro-[1,2,4]triazolo[5,1-*b*]quinazolin-8(4*H*)-one and 12-(4-hydroxyphenyl)-3,3-dimethyl-3,4,5,12-tetrahydrobenzo[4,5]imidazo[2,1-*b*]quinazolin-1(2*H*)-one catalyzed by NGPU with some of the other catalysts

Product	Catalyst (mol%)	Reaction conditions	Time (min)	Yield (%)
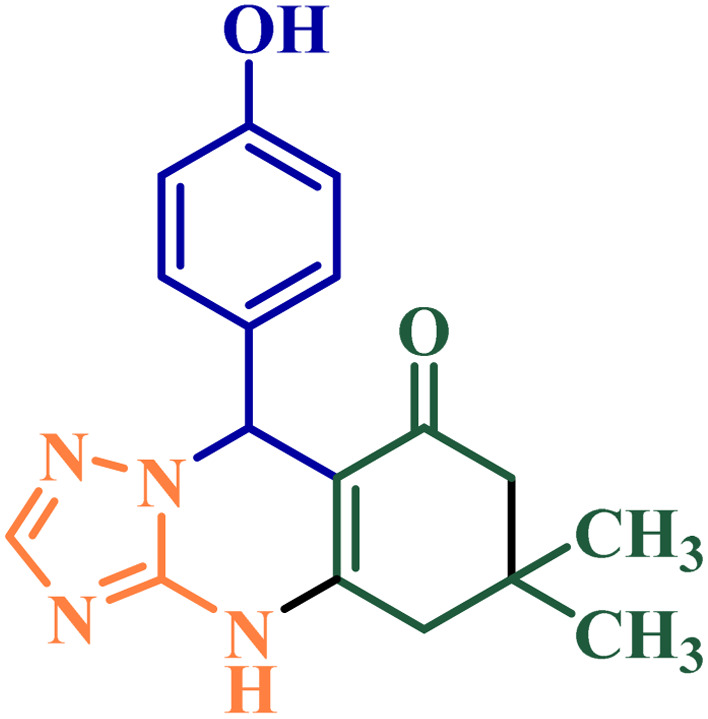	[DABCO](SO_3_H)_2_(HSO_4_)_2_ (0.02 mmol)^32^	Solvent free/100 °C	120	90
	NH_2_SO_3_H (50 mol%)^56^	CH_3_CN/reflux	60	89
	[C_4_(H-DABCO)_2_][HSO_4_]_4_ (16 mg)^33^	Solvent free/90 °C	28	87
	SBA–Pr–SO_3_H (5 mg)^34^	Solvent free/r.t	10	85
	[(DABCO)_2_C_3_H_5_OH] 2Cl (5.7 mol%)^31^	Solvent free/100 °C	40	95
	NGPU (2 : 1 : 5) (4 mol%)^a^	120 °C	18	95
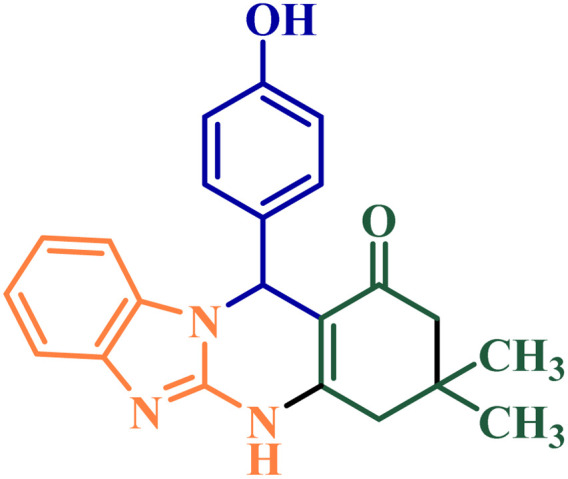	[DABCO](SO_3_H)_2_(HSO_4_)_2_ (0.02 mmol)^32^	Solvent free/100 °C	120	90
	NH_2_SO_3_H (0.05 mmol)^56^	CH_3_CN/reflux	20	90
	Na^+^-MMT-[pmim]HSO_4_ (50 mg)^35^	Solvent free/110 °C	60	91
	SBA–Pr–SO_3_H (5 mg)^34^	Solvent free/r.t	15	87
	Fe_3_O_4_@chitosan (2 mg)^36^	Ethanol/40 °C	135	95
	NGPU (2 : 1 : 5) (12 mol%)^a^	120 °C	14	99

a This work.

## Conclusions

In this study, a novel natural deep eutectic mixture (DEM) was prepared from glucose, urea, and pregabalin in three different ratios. The catalytic activity of the DEM was then investigated in a one-pot synthesis of triazolo[2,1-*b*]quinazolinone and benzazolo[2,1-*b*]quinazolinone derivatives. The study found that the optimal ratio of the DEM for the synthesis of triazolo/benzazolo[2,1-*b*]quinazolinone was 2 : 1 : 5 (NGPU), which was named after the components used in its preparation. This finding highlights the importance of optimizing the ratio of the components in the preparation of the DEM for specific applications.

The reaction was also tested under conditions without a catalyst, and the results were compared with the results obtained using the NGPU catalyst. The current protocol is based on the use of readily available natural components for the synthesis of the catalyst, simple and direct work-up procedures, use of a reusable and biodegradable catalyst, short reaction times, high purity, and high yields of the desired product, all major advantages of this protocol. Further studies are currently underway in our laboratory to investigate additional responses that may be mediated by this natural deep eutectic mixture.

## Experimental

### Material

Chemicals were purchased from the Fluka, Merck and Aldrich Chemical Companies. Yields refer to the isolated products. Products were characterized by their physical constants and spectral analysis techniques. All the reactions were monitored by thin layer chromatography (TLC) on silica-gel polygram SILG/UV 254 plates using UV light as a means of detection.

### Instrumentation

FT-IR spectra were recorded on a VERTEX 70 (Brucker, Germany) instrument using KBr pellets for samples ranging from 4000 to 400 cm^−1^. ^1^H NMR and ^13^C NMR were performed on 400 and 500 MHz Bruker Avance in DMSO-d_6_ using TMS as the internal standard. Melting points were determined using an IA9100 electric heating apparatus (Germany). Thermogravimetric analysis (TGA), derivative thermogravimetry (DTG) and differential thermal analysis (DTA) were performed on a Polymer Laboratories PL-TGA thermal analyzer. The sample was heated from 25 to 600 °C with a gradient of 20 °C min^−1^ under an Ar atmosphere (America). Refractive index measurements of the samples were made at a wavelength of 589 nm using a calibrated semi-automatic digital refractometer (model CETI) with a manufacturer-specified uncertainty of 0.0002 *n*_D_ (Germany). The device was calibrated with HPLC water and acetone before use. According to various measurements, the refractive index measurement uncertainty was estimated to be 0.0005 *n*_D_. The instrument temperature was held constant and measured with an uncertainty of 0.02 K. The absorption spectra were recorded over the wavelength range 200–800 nm using a Cary double beam UV-visible spectrophotometer (model 100) at room temperature (Australia). The uncertainty in the measured wavelength was found to be 0.1 nm. Quartz cells were used for measurements in a solution vial = 1 cm.

#### Preparation of the aimed natural deep eutectic mixture (NGPU)

**Scheme 4:**
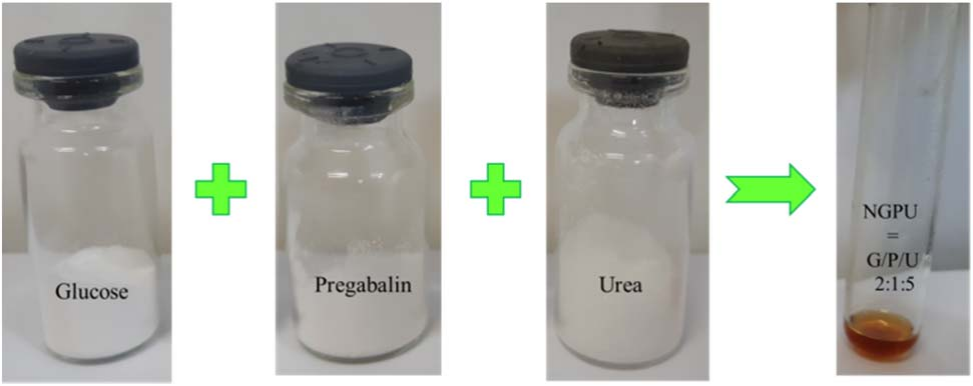
Preparation of NGPU.

A mixture of glucose, pregabalin and urea with a molar ratio of 2 : 1 : 5 was heated in a test tube at 110 °C under constant stirring for 30 min. During this period of time, the requested deep eutectic mixture (NGPU) appeared as a clear and homogeneous liquid and was collected without further purification (Scheme 4). The obtained product was characterized using FT-IR, TGA, DTG, DTA, RI and UV-vis spectra techniques.

#### Synthesis of triazoloquinazolinone derivatives: general procedure

1,3-Cyclohexanedione derivative (1 mmol), aldehyde (1 mmol), 3-amino-1,2,4-triazole (1 mmol) and NGPU (G/P/U; 2 : 1 : 5) (8 mg, 4 mol%) were mixed and stirred at 120 °C. The reaction was followed-up by TLC [EtOAc: *n*-hexane (3 : 7)]. After completion of the reaction, the solid product was separated by the addition of water (20 mL), which solvates the catalyst, and filtration. Washing with water (10 mL) and recrystallization from ethanol (if needed) led to the requested product with a high purity. The spectral data of the new compounds are as follows.

##### 9-(2-Methyl)-5,6,7,9-tetrahydro-[1,2,4]triazolo[5,1-*b*] quinazolin-8(4*H*)-one (19a)

FT-IR (KBr): *ν* = 3427, 3089, 2923, 1683, 1583, 1473, 1415, 1365, 1192, 738 cm^−1^; ^1^H NMR (400 MHz, DMSO-d_6_) *σ*: 1.89–2.02 (m, 2H), 2.23–2.30 (m, 2H), 2.58 (s, 3H), 2.58–2.72 (m, 2H), 6.46 (s, 1H), 7.02–7.16 (m, 4H), 7.65 (s, 1H), 11.14 (s, 1H, NH) ppm; ^13^C NMR (125 MHz, DMSO-d_6_) *σ*: 19.4, 21.2, 26.8, 36.8, 54.7, 107.7, 126.8, 127.3, 128.0, 130.5, 136.2, 140.8, 146.9, 150.4, 153.1, 193.8 ppm.

##### 9-(2-Bromophenyl)-5,6,7,9-tetrahydro-[1,2,4]triazolo[5,1-*b*] quinazolin-8(4*H*)-one (20a)

FT-IR (KBr): *ν* = 3430, 3087, 2917, 1644, 1415, 1365, 1191, 752, 534 cm^−1^; ^1^H NMR (500 MHz, DMSO-d_6_) *σ*: 1.89–1.94 (m, 2H), 2.15–2.22 (m, 2H), 2.63–2.64 (m, 2H), 6.55 (s, 1H), 7.09–7.36 (m, 3H), 7.42–7.80 (m, 2H), 11.20 (s, 1H, NH) ppm; ^13^C NMR (125 MHz, DMSO-d_6_) *σ*: 20.7, 26.5, 36.4, 39.1, 39.3, 58.0, 106.0, 127.9, 129.6, 132.8, 146.6, 150.1, 153.1, 193.2 ppm.

##### 9-(4-(Methylthio)phenyl)-5,6,7,9-tetrahydro-[1,2,4]triazolo [5,1-*b*]quinazolin-8(4*H*)-one (21a)

FT-IR (KBr): *ν* = 3424, 3088, 2912, 1645, 1587, 1480, 1363, 1186, 732 cm^−1^; ^1^H NMR (500 MHz, DMSO-d_6_) *σ*: 1.87–1.94 (m, 2H), 2.19–2.26 (m, 2H), 2.40 (s, 3H), 2.60–2.63 (m, 2H), 6.15 (s, 1H), 7.10–7.14 (m, 4H), 7.65 (s, 1H), 11.10 (s, 1H, NH) ppm; ^13^C NMR (125 MHz, DMSO-d_6_) *σ*: 14.6, 39.1, 39.3, 57.3, 106.5, 125.7, 127.6, 137.6, 146.7 ppm.

#### Synthesis of benzimidazoquinazolinone derivatives: general procedure

1,3-Cyclohexanedione derivative (1 mmol), aldehyde (1 mmol), 2-aminobenzimidazole (1 mmol) and NGPU (G/P/U; 2 : 1 : 5) (16 mg, 8 mol%) were mixed in a round bottom flask (50 mL) and stirred at 120 °C. Upon completion of the reaction, which was determined by TLC [EtOAc: *n*-hexane (3 : 7)], water (20 mL) was added to the mixture; the solid product (which is not soluble in water) was filtered, washed with water (10 mL) and dried. The spectral data of the new compounds are as follows.

##### 12-(2-Methyl)-3,4,5,12-tetrahydrobenzo[4,5]imidazo[2,1-*b*] quinazolin-1(2*H*)-one (11b)

FT-IR (KBr): *ν* = 3442, 3044, 2903, 1649, 1618, 1567, 1459, 1368, 737 cm^−1^; ^1^H NMR (500 MHz, DMSO-d_6_) *σ*: 1.81–1.95 (m, 2H), 2.16–2.26 (m, 2H), 2.57 (s, 3H), 2.64–2.68 (m, 2H), 6.47 (s, 1H), 6.86–7.35 (m, 8H), 11.05 (s, 1H, NH) ppm; ^13^C NMR (125 MHz, DMSO-d_6_) *σ*: 18.9, 20.8, 26.6, 36.5, 39.1, 39.3, 109.4, 117.0, 120.5, 121.8, 126.3, 127.4, 130.3, 132.1, 135.3, 141.9, 145.1, 152.1, 193.2 ppm.

##### 12-(2-Bromophenyl)-3,4,5,12-tetrahydrobenzo[4,5]imidazo [2,1-*b*]quinazolin-1(2*H*)-one (12b)

FT-IR (KBr): *ν* = 3453, 3041, 2957, 1654, 1616, 1570, 1445, 1368, 746, 543 cm^−1^; ^1^H NMR (500 MHz, DMSO-d_6_) *σ*: 1.84–1.96 (m, 2H), 2.05–2.26 (m, 2H), 2.65–2.68 (m, 2H), 6.60 (s, 1H), 6.91–7.49 (m, 8H), 11.20 (s, 1H, NH) ppm; ^13^C NMR (125 MHz, DMSO-d_6_) *σ*: 20.8, 26.7, 30.7, 36.5, 39.0, 79.2, 109.7, 117.1, 120.5, 128.1, 129.6, 132.1, 132.9, 141.8, 144.9, 152.7, 192.8 ppm.

##### 12-(Pyridin-4-yl)-3,4,5,12-tetrahydrobenzo[4,5]imidazo[2,1-*b*] quinazolin-1(2*H*)-one (14b)

FT-IR (KBr): *ν* = 3423, 3033, 2955, 1647, 1613, 1567, 1428, 1373, 1193, 743 cm^−1^; ^1^H NMR (500 MHz, DMSO-d_6_) *σ*: 1.88–2.02 (m, 2H), 2.23–2.35 (m, 2H), 2.72 (t, *J* = 11.1, 2H), 6.50 (s, 1H), 6.97–7.07 (m, 1H), 7.09–7.12 (m, 1H), 7.26 (d, *J* = 7.9, 2H), 7.30–7.32 (m, 1H), 7.42 (d, *J* = 7.8, 1H), 8.45–8.47 (m, 2H), 11.28 (s, 1H, NH) ppm; ^13^C NMR (125 MHz, DMSO-d_6_) *σ*: 21.1, 27.0, 36.7, 53.6, 110.6, 110.3, 117.6, 121.2, 122.5, 122.6, 132.2, 142.4, 145.6, 150.0, 150.3, 153.6, 193.5 ppm.

## Conflicts of interest

There are no conflicts to declare.

## Supplementary Material

RA-013-D3RA05199D-s001
