# Supplementary material for: Facile synthesis of triazolo/benzazolo[2,1-b]quinazolinone derivatives catalyzed by a new deep eutectic mixture based on glucose, pregabalin and urea
Source: RSC Adv. 2023 Oct 27;13(45):31470–9. doi: 10.1039/d3ra05199d (PMC10603617; doi:10.1039/d3ra05199d)
Supplement: RA-013-D3RA05199D-s001 [file RA-013-D3RA05199D-s001.pdf]

Electronic Supplementary Material (ESI) for RSC Advances.
This journal is © The Royal Society of Chemistry 2023

Supporting Information

Facile synthesis of triazolo/benzazolo[2,1-*b*]quinazolinones derivatives catalyzed by a new deep eutectic mixture based on glucose, pregabalin and urea

Parissa Naddaf Rahro, Farhad Shirini *

Department of Chemistry, College of Science, University of Guilan, Rasht, zip code 41335, I.R. Iran

Post Box: 1914, I. R. Iran. Tel./Fax: +98 131 3233262, E-mail address: shirini@guilan.ac.ir

6,6-dimethyl-9-phenyl-5,6,7,9-tetrahydro-[1,2,4]triazolo[5,1-*b*]quinazolin-8(4*H*)-one (1a):

FT-IR (KBr, cm^{-1}): 3445, 2962, 1650, 1594, 1374, 1252, 721 cm^{-1}. **^{1}H NMR** (400 MHz, DMSO-d_6) δ: 0.95 (s, 3H, CH_3), 1.03 (s, 3H, CH_3), 2.05 (d, *J* =16 Hz, 1H, CH_2), 2.19 (d, *J* = 16 Hz, 1H, CH_2), 2.52-2.59 (m, 2H, CH_2), 6.19 (s, 1H, CH), 7.17-7.29 (m, 5H-Ar), 7.68 (s, 1H-Ar), 11.14 (s, 1H, NH); **^{13}C NMR** (100 MHz, DMSO-d_6): δ 26.77, 28.45, 32.16, 49.74, 57.89, 105.55, 126.92, 127.69, 128.23, 141.55, 146.82, 150.24, 150.39, 192.96 ppm.

9-(4-chlorophenyl)-6,6-dimethyl-5,6,7,9-tetrahydro-[1,2,4]triazolo[5,1-*b*]quinazolin-8(4*H*)-one (2a):

FT-IR (KBr, cm^{-1}): 3436, 3094, 2961, 1649, 1578, 1367, 1254, 760 cm^{-1}; **^{1}H NMR** (400 MHz, DMSO-d_6) δ: 0.71 (s, 3H, CH_3), 0.79 (s, 3H, CH_3), 1.81 (d, *J* = 16 Hz, 1H, CH_2), 1.95 (d, *J* = 16 Hz, 1H, CH_2), 2.25-2.34 (m, 2H, CH_2), 5.97 (s, 1H, CH), 6.95 (d, *J* = 8 Hz, 2H-Ar), 7.09 (d, *J* = 8 Hz, 2H-Ar), 7.45 (s, 1H-Ar), 10.95 (s, 1H, NH); **^{13}C NMR** (100 MHz, DMSO-d_6): δ 26.87, 28.36, 32.17, 49.71, 57.34, 105.16, 128.25, 128.86, 132.26, 140.49, 146.76, 150.17, 150.55, 192.98 ppm.

9-(2-Chlorophenyl)-6,6-dimethyl-5,6,7,9-tetrahydro-[1,2,4]triazolo[5,1-*b*]quinazolin-8(4*H*)-one (3a):

FT-IR (KBr, cm^{-1}): 3224, 3085, 2923, 1643, 1573, 1365, 1257, 748 cm^{-1}; **^{1}H NMR** (250 MHz, DMSO-d_6) δ: 0.97(s, 3H, CH_3), 1.02 (s, 3H, CH_3), 2.04-2.05 (m, 1H, CH_2), 2.19 (d, 1H, *J* = 17.5 Hz, CH_2), 2.51–2.60 (m, 2H, CH_2), 6.55 (s, 1H, CH), 7.21–7.36 (m, 4H, ArH), 7.65 (s, 1H, =CH), 11.20 (s, 1H, NH); **^{13}C NMR** (62.9 MHz, DMSO-d_6) δ: 25.66, 27.38, 30.95, 48.62, 55.23, 103.38, 116.89, 125.99, 128.26, 128.43, 137.05, 145.72, 148.91, 149.90, 191.71 ppm.

9-(2,4-Dichlorophenyl)-6,6-dimethyl-5,6,7,9-tetrahydro-[1,2,4]triazolo[5,1-b]quinazolin-8(4H)-one (4a):

FT-IR (KBr, cm^{-1}): 3090, 2941, 1643, 1610, 1438, 1261,839 cm^{-1}; **^{1}H NMR** (300 MHz, DMSO-d_6) δ: 0.99 (s, 3H, CH_3), 1.09 (s, 3H, CH_3), 2.08 (q, *J* = 14.17, 23.03 Hz, 2H, CH_2), 2.49 (s, 2H, CH_2), 6.48 (s, 1H, CH), 7.22–7.31 (m, 2H, Ar-H), 7.45 (s, 1H, Ar-H), 7.59 (s, 1H, Ar-H), 10.92 (s, 1H, NH); **^{13}C NMR** (75 MHz, $CDCl_3$) δ: 13.11, 15.71, 59.48, 60.52, 110.18, 123.35, 125.12, 129.47, 141.86, 161.39 ppm.

9-(4-bromophenyl)-6,6-dimethyl-5,6,7,9-tetrahydro-[1,2,4]triazolo[5,1-*b*]quinazolin-8(4*H*)-one (5a):

FT-IR (KBr, cm^{-1}): 3436, 3090, 2965, 1650, 1584, 1367, 1255, 759 cm^{-1}; **^{1}H NMR** (400 MHz, DMSO-d_6) δ: 0.95 (s, 3H, CH_3), 1.03 (s, 3H, CH_3), 2.05 (d, *J* = 16 Hz, 1H, CH_2), 2.19 (d, *J* = 16 Hz, 1H, CH_2), 2.54-2.58 (m, 2H, CH_2), 6.19 (s, 1H, CH), 7.13 (d, *J* = 8 Hz, 2H-Ar), 7.47 (d, *J* = 8 Hz, 2H-Ar), 7.70 (s, 1H-Ar), 11.19 (s, 1H, NH); **^{13}C NMR** (100 MHz, DMSO-d_6): δ 26.87, 28.36, 32.18, 49.71, 57.43, 105.12, 120.85, 129.22, 131.18, 140.91, 146.75, 150.18, 150.56, 192.99 ppm.

6,6-dimethyl-9-(4-nitrophenyl)-5,6,7,9-tetrahydro-[1,2,4]triazolo[5,1-*b*]quinazolin-8(4*H*)-one (6a):

FT-IR (KBr, cm^{-1}): 3412, 3090, 2964, 1646, 1579, 1351, 1253, 729 cm^{-1}; **^{1}H NMR** (400 MHz, DMSO-d_6) δ: 0.95 (s, 3H, CH_3), 1.03 (s, 3H, CH_3), 2.05(d, *J* = 16Hz, 1H, CH_2), 2.19 (d, *J* = 16Hz, 1H, CH_2), 2.49 (d, *J* = 16Hz, 1H, CH_2), 2.56 (d, *J* = 16Hz, 1H, CH_2), 6.36 (s, 1H, CH), 7.47 (d, J = 8Hz, 2H-Ar), 7.73 (s, 1H-Ar), 8.14 (d, *J* = 8Hz, 2H-Ar), 11.31 (s, 1H, NH); **^{13}C NMR** (100 MHz, DMSO-d_6) δ: 26.92, 28.26, 32.21, 49.63, 57.49, 104.72, 123.55, 128.46, 146.81, 146.89, 148.38, 150.40, 150.97, 193.01 ppm.

6,6-dimethyl-9-(3-nitrophenyl)-5,6,7,9-tetrahydro-[1,2,4]triazolo[5,1-b]quinazolin-8(4H)-one (7a):

FT-IR (KBr, cm^{-1}): 3436, 3082, 2960, 1645, 1580, 1367, 1256, 732 cm^{-1}. **^{1}H NMR** (400 MHz, DMSO-d_6) δ: 0.756 (s, 3H, CH_3), 0.829 (s, 3H, CH_3), 1.86 (d, *J* = 16 Hz, 1H, CH_2), 1.99 (d, *J* = 16 Hz, 1H, CH_2), 2.28-2.40 (m, 2H, CH_2), 6.20 (s, 1H, CH), 7.37-7.44 (m, 2H-Ar), 7.52 (s, 1H-Ar), 7.84- 7.90 (m, 2H-Ar), 11.09 (s,1H, NH); **^{13}C NMR** (100 MHz, DMSO-d_6): δ 26.85, 28.31, 32.23, 49.65, 57.39, 104.59, 121.66, 122.81, 130.04, 133.71, 143.45, 146.77, 147.58, 150.42, 151.13, 193.09 ppm.

9-(4-methoxyphenyl)-6,6-dimethyl-5,6,7,9-tetrahydro-[1,2,4]triazolo[5,1-*b*]quinazolin-8(4*H*)-one (8a):

FT-IR (KBr, cm^{-1}): 3436, 2961, 1649, 1580, 1367, 1246, 745 cm^{-1}. **1H NMR** (400 MHz, DMSO-d_6) δ: 0.75 (s, 3H, CH_3), 0.82 (s, 3H, CH_3), 1.83 (d, *J* = 16 Hz, 1H, CH_2), 1.98 (d, *J* = 16 Hz, 1H, CH_2), 2.28-2.37 (m, 2H, CH_2), 3.47 (s, 3H, OCH_3), 5.93 (s, 1H, CH), 6.60 (d, *J* = 8 Hz, 2H-Ar), 6.87 (d, *J* = 8 Hz, 2H-Ar), 7.45 (s, 1H-Ar), 10.87 (s,1H, NH); **^{13}C NMR** (100 MHz, DMSO-d_6): δ 26.79, 28.49, 32.15, 49.78, 54.99, 57.30, 105.71, 113.54, 128.09, 133.82, 146.72, 150.14, 158.66, 192.94 ppm.

9-(4-hydroxyphenyl)-6,6-dimethyl-5,6,7,9-tetrahydro-[1,2,4]triazolo[5,1-*b*]quinazolin- 8(4*H*)-one (9a):

FT-IR (KBr, cm^{-1}): 3348, 3096, 2932, 1631, 1587, 1370, 1269, 731 cm^{-1}; **^{1}H NMR** (400 MHz, DMSO-d_6) δ: 0.96 (s, 3H, CH_3), 1.03 (s, 3H, CH_3), 2.05 (d, *J* = 16 Hz, 1H, CH_2), 2.18 (d, *J* = 16 Hz, 1H, CH, CH_2), 2.57-2.68 (m, 2H, CH_2), 6.08 (s, 1H, CH), 6.62 (d, *J* = 8 Hz, 2H-Ar), 6.96 (d, *J* = 8 Hz, 2H-Ar), 7.65 (s, 1H-Ar), 9.39 (s, 1H, OH or NH), 11.04 (s, 1H, OH or NH); **^{13}C NMR** (100 MHz, DMSO-d_6) δ: 26.79, 28.51, 32.14, 49.80, 52.99, 57.33, 105.86, 114.84, 132.21, 128.07, 146.69, 149.83, 150.03, 156.87, 192.94 ppm.

9-(4-hydroxy-3-methoxyphenyl)-6,6-dimethyl-5,6,7,9-tetrahydro-[1,2,4]triazolo[5,1-*b*]quinazolin-8(4*H*)-one (10a):

FT-IR (KBr, cm^{-1}): 3081, 2968, 1655, 1589, 1375, 1242, 764 cm^{-1}; **^{1}H-NMR** (400 MHz, DMSO-d_6) δ: 1.01 (s, 3H,CH_3), 1.08 (s, 3H, CH_3), 2.11 (q, *J* = 13.62, 22.79 Hz, 2H, CH_2), 2.43 (s, 2H, CH_2) 3.70 (s, 3H, OCH_3), 6.71 (s, 1H, CH), 7.11-7.20 (m, 2H, Ar-H), 7.37 (s, 1H, Ar-H), 7.49 (s, 1H, Ar-H), 11.21 (s, 1H, NH); **^{13}C NMR** (75

MHz, $CDCl_3$) δ: 25.37, 27.56, 30.72, 48.10, 49.33, 101.17, 122.48, 126.51, 131.77, 133.12, 146.57, 149.44, 155.83, 157.46, 189.74 ppm.

6,6-dimethyl-9-(p-tolyl)-5,6,7,9-tetrahydro-[1,2,4]triazolo[5,1-*b*]quinazolin-8(4*H*)-one (11a):

FT-IR (KBr, cm^{-1}): 3380–3022, 2957, 2922, 1711, 1610, 1461 cm^{-1} ;^{1}**H NMR** (400 MHz, DMSO-d_6) δ: 0.98 (s, 3H, CH_3), 1.11 (s, 3H, CH_3), 2.17 (d, J = 5.7 Hz, 1H, CH_2), 2.24 (d, J = 5.7 Hz, 1H, CH_2), 2.38 (s, 3H, CH_3), 2.62–2.70 (m, 2H, CH_2), 6.09 (s, 1H, CH), 7.03 (s, 2H-Ar), 7.03 (s, 2H-Ar), 7.70 (s, 1H-Ar), 11.05 (s, 1H, NH); ^{13}**C NMR** (100 MHz, DMSO-d_6) δ: 22.3, 24.8, 29.2, 48.5, 51.6, 57.2, 108.1, 126.0 ,128.6, 129.9, 143.6, 144.1, 149.0, 154.5, 192.9 ppm.

6,6-Dimethyl-9-(o-tolyl)-5,6,7,9-tetrahydro-[1,2,4]triazolo[5,1-*b*]quinazolin-8(4*H*)-one (12a):

FT-IR (KBr, cm^{-1}): 3085, 2925, 1643, 1581, 1365, 1257, 748 cm^{-1}; ^{1}**H NMR** (250 MHz, DMSO-d_6) δ: 0.97(s, 3H, CH_3), 1.03 (s, 3H, CH_3), 2.12 (q, 2H, J = 17.5 Hz, CH_2), 2.48–2.60 (m, 5H, CH_3 and CH_2), 6.42 (s, 1H, CH), 6.95–7.09 (m, 4H-Ar), 7.63 (s, 1H-Ar), 11.09 (s, 1H, NH); ^{13}**C NMR** (62.9 MHz, DMSO-d_6) δ: 18.89, 26.83, 28.48, 32.18, 49.73, 54.12, 106.14, 126.18, 126.64, 127.43, 129.96, 135.56, 140.32, 146.55, 149.87, 150.42, 193.00 ppm.

9-(phenyl)-5,6,7,9-tetrahydro-1,2,4-triazolo[5,1-*b*]quinazolin-8(4*H*)-one (13a):

FT-IR (KBr, cm^{-1}): 3344, 2990, 1715, 1638, 1584, 1549, 1412, 1365 cm^{-1}; ^{1}**H NMR** (300 MHz, DMSO-d_6) δ: 1.85-2.66 (m, 6H, $3CH_2$), 6.22 (s, 1H, CH), 6.91-7.30 (m, 5H-Ar), 7.69 (s, 1H, CH), 11.19 (br. s, 1H, NH); ^{13}**C NMR** (75 MHz, DMSO-d_6) δ: 21.12, 26.85, 36.77, 58.18, 107.05, 127.45, 150.82, 128.15, 128.72, 128.87, 141.97, 147.22, 150.51, 153.03, 193.76 ppm.

9-(4-chlorophenyl)-5,6,7,9-tetrahydro-[1,2,4]triazolo[5,1-*b*]quinazolin-8(4*H*)-one (14a):

^{1}**H NMR** (400 MHz, DMSO-d_6) δ: 1.88-1.99 (m, 2H CH_2), 2.20- 2.34 (m, 2H, CH_2), 2.64-2.71 (m, 2H, CH_2), 6.24 (s, 1H, CH), 7.24 (d, J = 8.4 Hz, 2H, Ar-H), 7.35 (d, J = 8.8 Hz, 2H, Ar-H), 7.71 (s, 1H, Ar-H), 11.20 (s, NH, 1H); ^{13}**C NMR** (100 MHz, DMSO-d_6) δ: 20.6, 26.3, 36.2, 57.17, 106.1, 2 x 128.2, 2 x 128.9, 132.2, 140.4, 146.6, 150.1, 152.7, 193.2 ppm.

9-(3,4,5-Trimethoxyphenyl)-5,6,7,9-tetrahydro-1,2,4-triazolo[5,1-*b*]quinazolin-8(4H)-one (15a):

FT-IR (KBr, cm^{-1}): 3433, 2956, 2924, 2834, 1645, 1595, 1507, 1460, 1416, 1328, 1239, 1128, 1009, 888, 832, 580 cm^{-1}; ^{1}**H NMR** (400 MHz, DMSO-d_6) δ: 1.90–2.03 (m, 2H, CH), 2.28–2.31 (m, 2H, CH), 2.60–2.57 (m, 2H, CH), 3.61 (s, 3H, OCH3), 3.71 (s, 6H, OCH3), 6.81 (s, 1H, CH), 6.48 (s, 2H, ArH), 7.70 (s, 1H, ArH), 11.13 (s, 1H, NH) ppm; ^{13}**C NMR** (100 MHz, DMSO-d_6) δ: 20.7, 26.4, 36.3, 55.8, 57.6, 59.8, 104.3, 106.1, 136.9, 146.6, 149.9, 152.7, 193.4ppm.

9-(4-bromophenyl)-5,6,7,9-tetrahydro-[1,2,4]triazolo[5,1-*b*]quinazolin-8(4*H*)-one (16a):

FT-IR (KBr, cm^{-1}): 3429, 3129, 2883, 1648, 1578, 621 cm^{-1}; ^{1}**H NMR** (400 MHz, DMSO-d_6) δ: 1.89–2.01 (m, 2H, CH_2), 2.22–2.34 (m, 2H, CH_2), 2.61–2.71 (m, 2H, CH_2), 6.23 (s, 1H, CH), 7.18 (d, J = 8.4 Hz, 2H-Ar), 7.49

(d, J = 8.4 Hz, 2H-Ar), 7.71 (s, 1H-Ar), 11.21 (s, 1H, NH); **^{13}C NMR** (100 MHz, DMSO-d_6) δ: 21.1, 26.8, 36.7, 44.4, 57.7, 106.6, 121.3, 129.7, 131.6, 141.3, 147.1, 150.6, 153.2, 193.8 ppm.

9-(4-methyl-phenyl)-5,6,7,9-tetrahydro-1,2,4-triazolo[5,1-*b*]quinazolin-8(4H)-one (17a):

FT-IR (KBr, cm^{-1}): 3425, 2922, 2990, 1643, 1575, 1550, 1412, 1361 cm^{-1}; **^{1}H NMR** (300 MHz, DMSO-d_6) δ: 1.88-2.66 (m, 6H, 3CH_2), 2.08 (s, 3H, CH_3), 6.18 (s, 1H, CH), 7.07 (m, 4H-Ar), 7.67 (s, 1H, CH), 11.14 (br. s, 1H, NH); **^{13}C NMR** (75 MHz, DMSO-d_6) δ: 21.08, 21.14, 26.84, 36.78, 57.89, 107.17, 127.34, 129.24, 137.41, 139.16, 147.18, 150.42, 152.85, 193.73 ppm.

9-(3-nitro-phenyl)-5,6,7,9-tetrahydro-1,2,4-triazolo[5,1-*b*]quinazolin-8(4H)-one (18a):

FT-IR (KBr, cm^{-1}): 3410, 3220, 2930, 1620, 1557, 1521, 1465, 1405, 1347 cm^{-1}; ^{1}H NMR (300 MHz, DMSO-d_6) δ:1.93- 2.02 (m, 2H, CH_2), 2.20-2.33 (m, 2H, CH_2), 2.66-2.75 (m, 2H, CH_2), 6.42 (s, 1H, CH), 7.57-7.68 (m, 2H, CH-Ar), 7.74 (s, 1H-Ar), 8.07-8.12 (m, 2H-Ar), 11.33 (br. s, 1H, NH); **^{13}C NMR** (75 MHz, DMSO-d_6) δ: 21.10, 26.92, 36.68, 57.77, 106.10, 122.25, 123.27, 130.49, 134.14, 143.88, 147.18, 148.09, 150.91, 153.73, 193.92 ppm.

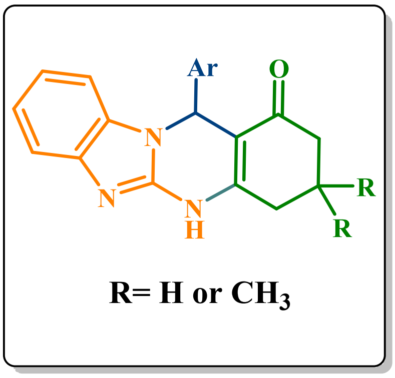

3,3-Dimethyl-12-phenyl-3,4,5,12-tetrahydrobenzimidazo[2,1-*b*]quinazolin-1(2*H*)-one (1b):

FT-IR (KBr, cm^{-1}): 1569, 1647, 2956, 3367 cm^{-1}. **^{1}H NMR** (300 MHz, DMSO-d_6) δ: 1.06 (s, 3H, CH_3), 1.17 (s, 3H, CH_3), 2.23-2.37 (dd, J = 16.43, 30.48 Hz, 2H, CH_2), 2.67 (s, 2H, CH_2), 6.49 (s, 1H, CH), 7.09-7.65 (m, 9H, Ar-H), 10.87 (s, 1H, NH). **^{13}C NMR** (75 MHz, DMSO-d_6) δ: 26.73, 26.43, 31.79, 35.92, 49.99, 58.15, 112.56, 119.77, 125.57, 125.67, 126.19, 127.44, 127.66, 128.13, 132.02, 132.87, 142.05, 158.48, 158.53, 162.57, 195.73 ppm.

3,3-Dimethyl-12-(4-chloro-phenyl)-3,4,5,12-tetrahydrobenzimidazo[2,1-*b*]quinazolin-1(2*H*)-one (2b):

FT-IR (KBr, cm^{-1}): 3413, 3065, 2965, 1767, 1606, 1571, 1478, 1344, 1021 cm^{-1}; **^{1}H NMR** (300 MHz, DMSO-d_6) δ: 0.96 (s, 3H, CH_3), 1.15 (s, 3H, CH_3), 2.24 (s, 2H, CH_2), 2.67 (s, 2H, CH_2), 4.84 (s, 1H, C-H), 7.19-7.54 (m, 2H), 7.68 (d, J = 8.4 Hz, 3H), 8.01-8.26 (m, 3H) 11.31 (brs, N-H, 1H) ppm. **^{13}C NMR** (75 MHz, DMSO-d_6) δ: 18.76, 27.12, 28.13, 34.65, 51.55, 115.82, 120.60, 122.98, 125.01, 127.09, 129.87, 133.65, 145.12 (two peaks), 146.42, 147.56, 152.26, 167.01, 196.50 ppm.

3,3-Dimethyl-12-(4-bromo-phenyl)-3,4, 5,12-tetrahydrobenzimidazo[2,1-*b*]quinazolin-1(2*H*)-one (3b):

FT-IR (KBr, cm^{-1}): 3232, 3048, 2932, 2863, 1648 cm^{-1}; **^{1}H NMR** (300 MHz, DMSO-d_6) δ: 0.94 (s, 3H, CH_3), 1.05 (s, 3H, CH_3), 2.00-2.30 (m, 2H, CH_2), 2.60-2.70 (m, 2H, CH_2), 6.45 (s, 1H, CH), 6.98 (t, *J* = 8.0 Hz, 1H, H-Ar), 7.06 (t, *J* = 8.0 Hz, 1H, H-Ar), 7.25-7.30 (m, 4H, H-Ar), 7.35-7.45 (m, 2H, H-Ar), 11.15 (s, 1H, NH); **^{13}C NMR** (75 MHz, DMSO-d_6) δ: 27.9, 32.1, 43.1, 52.4, 58.5, 112.0, 114.4, 118.2, 123.6, 123.1, 130.3, 139.2, 143.2, 144.8, 1463.3, 148.8, 154.6, 199.8 ppm.

3,3-dimethyl-12-(4-hydroxyphenyl)-3,4,5,12-tetrahydrobenzimidazo[2,1-*b*]quinazolin-1(2*H*)-one (4b):

FT-IR (KBr, cm^{-1}): 3448, 3255, 2890, 1641, 1612, 1589, 1566 cm^{-1}; **^{1}H NMR** (300 MHz, DMSO-d_6) δ: 0.95 (s, 3H, CH_3), 1.06 (s, 3H, CH_3), 2.04 (d, *J* = 8.94 Hz, 1H, CH_2), 2.24 (d, *J* = 9.24 Hz, 1H, CH_2), 2.50–2.73 (m, 2H, CH_2), 6.19 (s, 1H, CH), 6.50–7.50 (m, 8H, H-Ar), 9.32 (s, 1H, OH), 11.01 (brs, 1H, NH). **^{13}C NMR** (75 MHz, DMSO-d_6): δ: 23 26.2, 47.4, 53.9, 110.5, 115.6, 122.9, 130.6, 137.9, 141.5, 154.3, 156.3, 156.3, 197.6 ppm.

3,3-Dimethyl-12-(2,4-dichlorophenyl)-3,4,5,12-tetrahydrobenzimidazo[2,1-*b*]quinazolin-1(2*H*)-one (5b):

FT-IR (KBr, cm^{-1}): 3238, 3061, 2963, 2931, 1650, 1615, 1595, 1573, 1563, 1459, 1374, 1270, 737; **^{1}H NMR** (400 MHz, DMSO-d_6): δ = 0.95 (s, 3H, CH_3), 1.06 (s, 3H, CH_3), 2.04 (d, *J* = 16.0 Hz, 1H, H-4), 2.24 (d, *J* = 16.0 Hz, 1H, H′-4), 2.29 (d, *J* = 12.0 Hz, 1H, H-2), 2.33 (d, *J* = 16.0 Hz, 1H, H′-2), 6.65 (S, 1H, H-12), 6.96 (t, *J* = 8.0 Hz, 1H, Ar–H), 7.03 (s, 1H, Ar–H), 7.08-7.12 (m, 1H, Ar–H), 7.38 (s, 1H, Ar–H), 7.40 (s, 1H, Ar–H), 7.48 (d, *J* = 8.0 Hz, 1H, Ar–H), 11.25 (s, 1H, NH) ppm.

3,3-Dimethyl-12-(3,4,5-trimethoxyphenyl)-3,4,5,12-tetrahydrobenzimidazo[2,1-*b*]quinazolin-1(2*H*)-one (6b):

FT-IR (KBr, cm^{-1}): 3235, 3096, 2958, 2866, 1647 cm^{-1};**^{1}H NMR** (300 MHz, DMSO-d_6) δ: 1.00 (s, 3H, CH_3), 1.08 (s, 3H, CH_3), 2.11-2.28 (m, 2H, CH_2), 2.60-2.70 (m, 2H, CH_2), 3.66 (s, 9H, OCH_3), 6.36 (s, 1H, CH),6.64 (s, 2H-Ar), 7.00 (dt, 1H-Ar), 7.06 (dt, 1H-Ar), 7.37 (d, 1H-Ar), 7.42 (d, 1H-Ar), 10.90 (s, 1H, NH); **^{13}C NMR** (75 MHz, DMSO-d_6) δ: 26.5, 28.8, , 32.1, 40.0, 50.1, 54.1, 105.1, 106.3, 110.2, 116.9, 120.4, 121.7, 132.0, 137.0137.2, 142.0, 145.4, 150.5, 152.7, 192.5 ppm.

3,3-dimethyl-12-(4-methyl-phenyl)-3,4,5,12-tetrahydrobenzimidazo[2,1-*b*]quinazolin-1(2*H*)-one (7b):

FT-IR (KBr, cm^{-1}): 3245, 3040, 2952, 2895, 1648 cm^{-1}; **^{1}H NMR** (300 MHz, DMSO-d_6) δ: 0.94 (s, 3H, CH_3), 1.05 (s, 3H, CH_3), 2.10 (s, 3H, CH_3), 2.00-2.30 (m, 2H, CH_2), 2.50-2.65 (m, 2H, CH_2), 6.35 (s, 1H, CH), 6.95 (t, *J* = 7.6 Hz, 1H, H-Ar), 7.02-7.06 (m, 3H, H-Ar), 7.20-7.28 (m, 3H, H-Ar), 7.38 (d, *J* = 8.6 Hz, 1H, H-Ar), 11.06 (s, 1H, NH); **^{13}C NMR** (75 MHz, DMSO-d_6) δ: 16.9, 20.9, 26.8, 47.3, 53.5, 110.2, 115.4, 122.9, 129.1, 134.0, 134.1, 134.7, 138.1, 141.3, 152.8, 198.4 ppm.

3,3-Dimethyl-12-(2-methyl-phenyl)-3,4,5,12-tetrahydrobenzimidazo[2,1-*b*]quinazolin-1(2*H*)-one (8b):

FT-IR (KBr, cm^{-1}): 3540, 3095, 1642, 1610, 1563 cm^{-1}; **^{1}H NMR** (300 MHz, DMSO-d_6) δ: 0.92 (s, 3H, CH_3), 1.05 (s, 3H, CH_3), 2.13 (q, *J* = 16.23 Hz, 2H, CH_2), 2.56 (q, *J* = 16.35 Hz, 2H, CH_2), 2.64 (s, 3H, CH_3), 6.51 (s, 1H, CH), 6.89-7.38 (m, 8H-Ar), 11.15 (s, 1H, NH) ppm.

3,3-Dimethyl-12-(3-nitrophenyl)-3,4,5,12-tetrahydrobenzimidazo[2,1-*b*]quinazolin-1(2*H*)-one (9b):

FT-IR (KBr, cm^{-1}): 3394, 2970, 1660, 1648,1615, 1598 cm^{-1}; **^{1}H NMR** (300 MHz, DMSO-d_6): d 0.92 (s, 3H, CH_3), 1.08 (s, 3H, CH_3), 2.08 (d, 1H, J = 16.2 Hz, one proton of diastereotopic protons in CH_2), 2.29 (d, 1H, J = 16.2 Hz, one proton of diastereotopic protons in CH_2), 2.55– 2.72 (m, 2H, CH_2), 6.67 (s, 1H, CH), 6.98 (td, 1H, J = 7.8 Hz, 1.2, HAr), 7.08 (td, 1H, J = 7.1, 0.9 Hz, HAr), 7.31 (d, 1H, J= 7.5 Hz, HAr), 7.41 (d, 1H, J = 7.5 Hz, HAr), 7.57 (t, 1H, J = 7.8 Hz, HAr), 7.74 (d, 1H, J = 7.8 Hz, HAr), 8.03–8.08 (m, 1H, HAr), 8.29 (t, 1H, J = 1.8 Hz, HAr), 11.30 (br., 1H, NH) ppm.

12-phenyl-3,4,5,12-tetrahydrobenzo[4,5]imidazo[2,1-*b*]quinazolin-1(2*H*)-one (10b):

FT-IR (KBr, cm^{-1}): 1560, 1666, 2968, 3370 cm^{-1}; **^{1}H NMR** (300 MHz, DMSO-d_6) δ: 1.86-1.93 (m, 2H, CH_3), 2.23-2.29 (m, 2H, CH_3), 2.59-2.61 (d, J = 5.05 Hz, 2H, CH_2), 6.49 (s, 1H, CH), 7.09-7.90 (m, 9H, Ar-H), 11.11 (s, 1H, NH); **^{13}C NMR** (75 MHz, DMSO-d_6) δ: 18.55, 19.73, 26.39, 30.14, 35.97, 49.99, 58.15, 112.64, 119.67, 125.56, 125.67, 126.28, 127.34, 127.54, 128.22, 132.13, 132.89, 142.08, 159.05, 159.09, 162.74, 196.04 ppm.

12-(3,4,5-trimethoxyphenyl)-3,4,5,12-tetrahydrobenzo[4,5]imidazo[2,1-*b*]quinazolin-1(2H)-one (13b):

FT-IR (KBr, cm^{-1}): 3430, 2998, 2890, 1641, 1612, 1589, 1555 cm^{-1}; **^{1}H NMR** (400 MHz, DMSO-d_6) δ: 1.89-2.01 (m, 2H), 2.23-2.34 (m, 2H), 2.63-2.77 (m, 2H), 3.55 (s, 3H), 3.68 (s, 6H), 6.39 (s, 1H), 6.63 (s, 2H), 6.97-7.01 (m, 2H), 7.37 (d, J = 7.6 Hz, 1H), 7.45 (d, J = 7.6 Hz, 1H), 11.08 (s, 1H)); **^{13}C NMR** (100 MHz, DMSO-d_6) δ: 20.7, 26.5, 36.4, 53.9, 55.8, 59.8, 104.5, 107.2, 110.2, 116.8, 120.4, 121.7, 132.0, 136.8, 137.1, 141.8, 145.1, 152.6, 192.9 ppm.

12-(4-nitrophenyl)-3,4,5,12-tetrahydrobenzimidazo[2,1-*b*]quinazolin-1(2*H*)-one (15b):

FT-IR (KBr, cm^{-1}): 3223, 3040, 2949, 1646, 1515, 898, 822 cm^{-1}; **^{1}H NMR** (400 MHz, DMSO-d_6) δ: 1.80-1.97 (m, 2H, CH_2), 2.15-2.31 (m, 2H, CH_2), 2.65-2.68 (m, 2H, CH_2), 6.59 (s, 1H, CH), 6.92 (t, J = 8.0 Hz, 1H, ArH), 7.02 (t, J = 7.2 Hz, 1H, ArH), 7.17 (d, J = 7.6 Hz, 1H, ArH), 7.35 (d, J = 8.0 Hz, 1H, ArH), 7.56 (d, J = 8.8 Hz, 2H, ArH), 8.06 (d, J = 8.8 Hz, 2H, ArH), 11.25 (s, 1H, NH) ppm.

12-(4-fluorophenyl)-3,4,5,12-tetrahydrobenzimidazo[2,1-*b*]quinazolin-1(2*H*)-one (16b):

FT-IR (KBr, cm^{-1}): 3227, 3043, 2956, 1644, 1510, 890, 759 cm^{-1}; **^{1}H NMR** (400 MHz, DMSO-d_6) δ: 1.83-1.97 (m, 2H, CH_2), 2.15-2.31 (m, 2H, CH_2), 2.63-2.65 (m, 2H, CH_2), 6.41 (s, 1H, CH), 6.92 (t, 1H, J = 7.2 Hz, ArH), 6.99-7.04 (m, 3H, ArH), 7.20 (d, 1H, J = 7.6 Hz, ArH), 7.31-7.34 (m, 3H, ArH), 11.10 (s, 1H, NH) ppm.

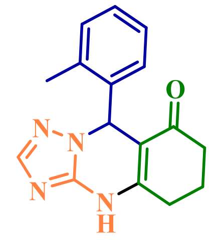
O
N
N
N
N
H

20a

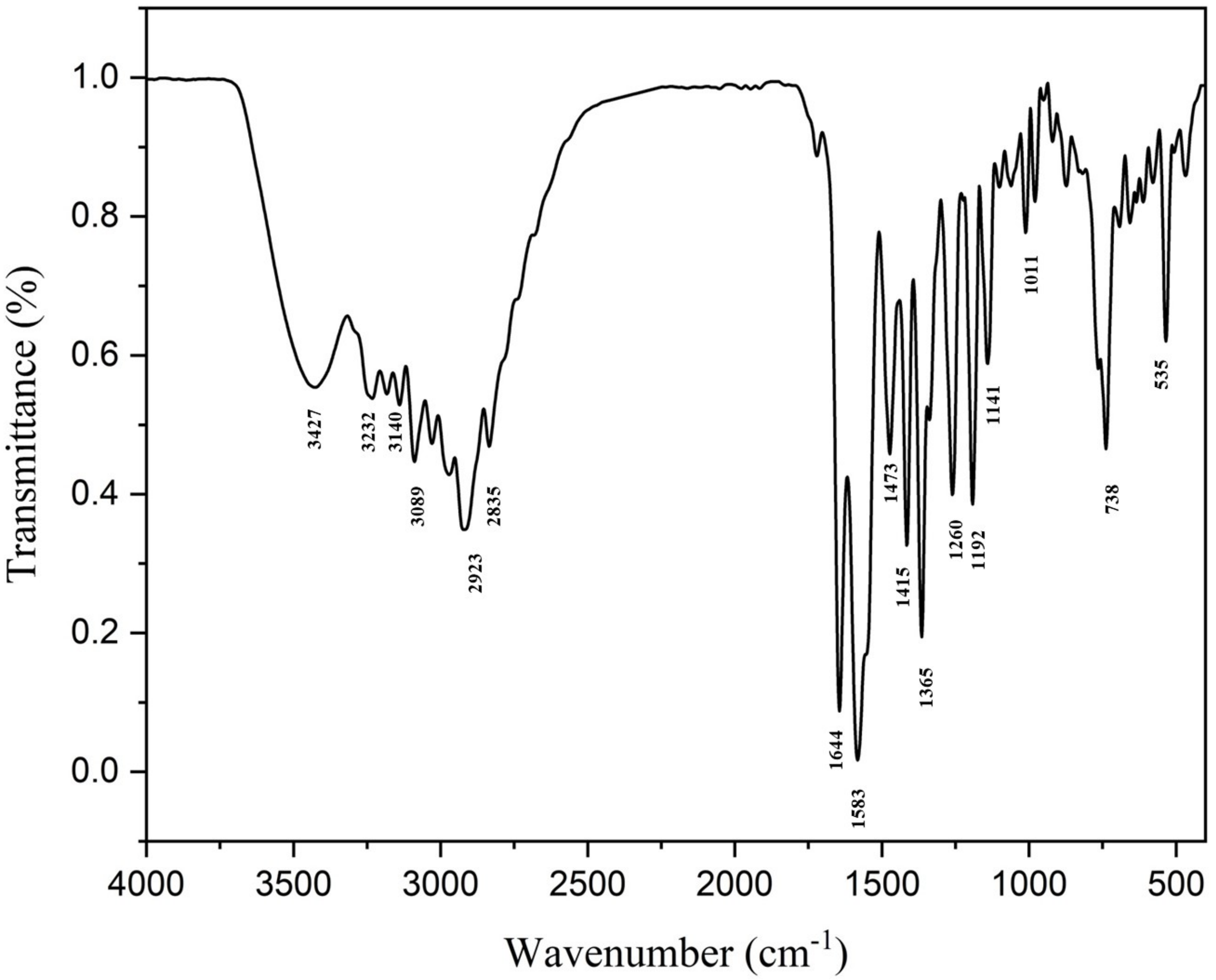
1.0
0.8
0.6
0.4
0.2
0.0
Transmittance (%)
3427
3232
3140
3089
2923
2835
1644
1583
1473
1415
1365
1260
1192
1141
1011
738
535
4000
3500
3000
2500
2000
1500
1000
500
Wavenumber (cm-1)

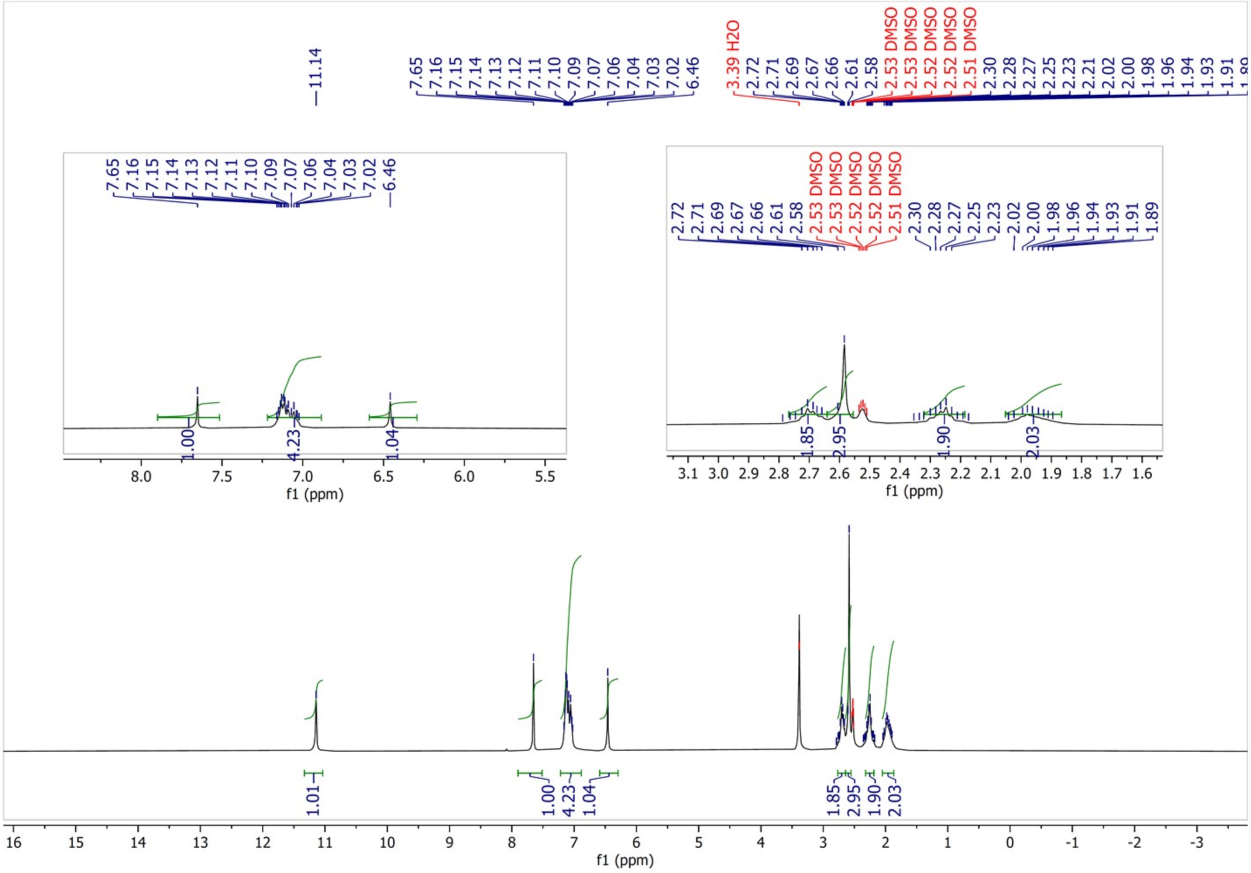
11.14
7.65 7.16 7.15 7.14 7.13 7.12 7.11 7.10 7.09 7.07 7.06 7.04 7.03 7.02 6.46
3.39 H2O
2.72 2.71 2.69 2.67 2.66 2.61 2.58
2.53 DMSO 2.53 DMSO 2.52 DMSO 2.52 DMSO 2.51 DMSO
2.30 2.28 2.27 2.25 2.23 2.21 2.02 2.00 1.98 1.96 1.94 1.93 1.91
1.00 4.23 1.04
1.85 2.95 1.90 2.03
1.01 1.00 4.23 1.04 1.85 2.95 1.90 2.03
f1 (ppm)

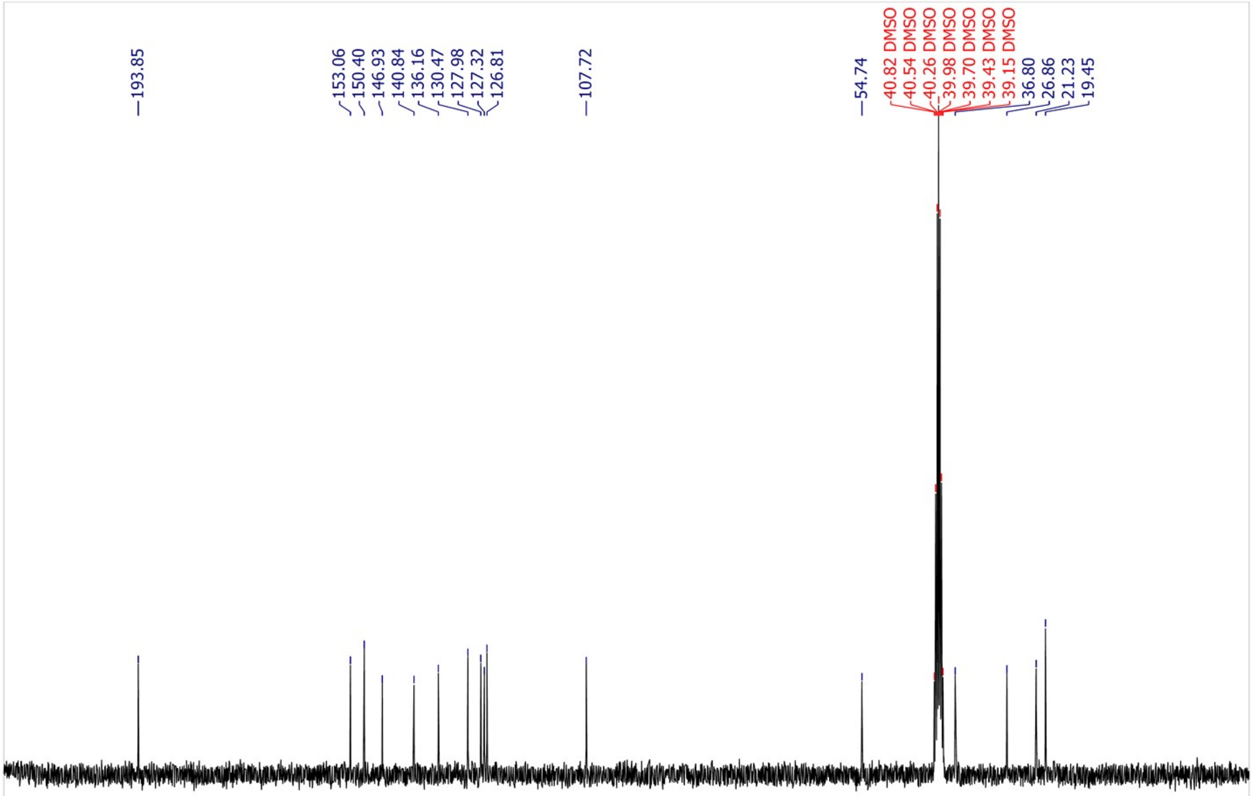
193.85
153.06 150.40 146.93 140.84 136.16 130.47 127.98 127.32 126.81
107.72
54.74
40.82 DMSO 40.54 DMSO 40.26 DMSO 39.98 DMSO 39.70 DMSO 39.43 DMSO 39.15 DMSO
36.80 26.86 21.23 19.45
f1 (ppm)

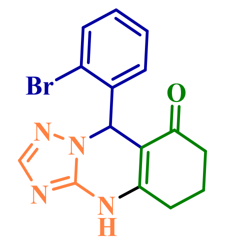
Br
O
N
N
N
N
H

21a

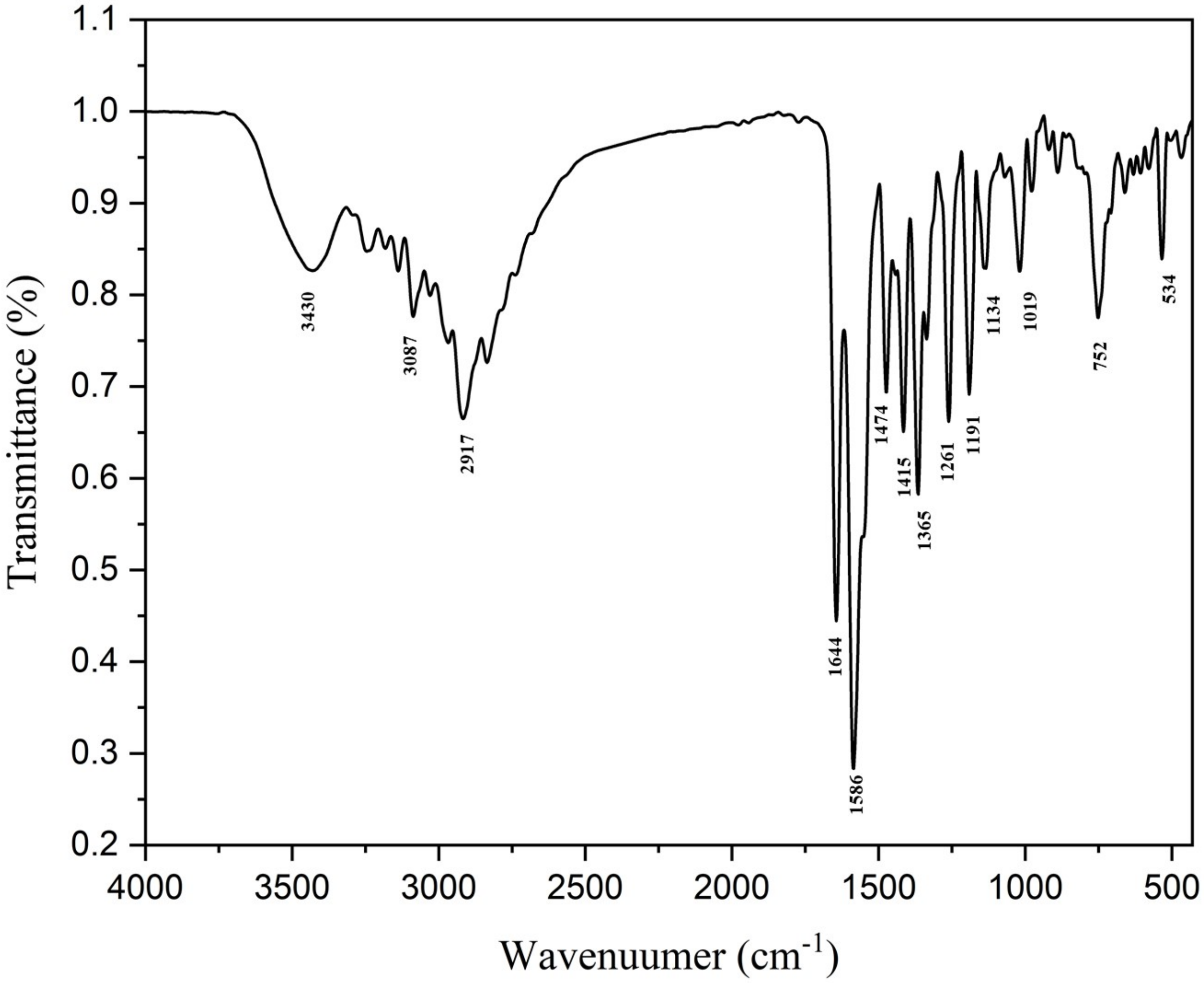
Transmittance (%)
Wavenuumer (cm-1)
1.1
1.0
0.9
0.8
0.7
0.6
0.5
0.4
0.3
0.2
4000
3500
3000
2500
2000
1500
1000
500
3430
3087
2917
1644
1586
1474
1415
1365
1261
1191
1134
1019
752
534

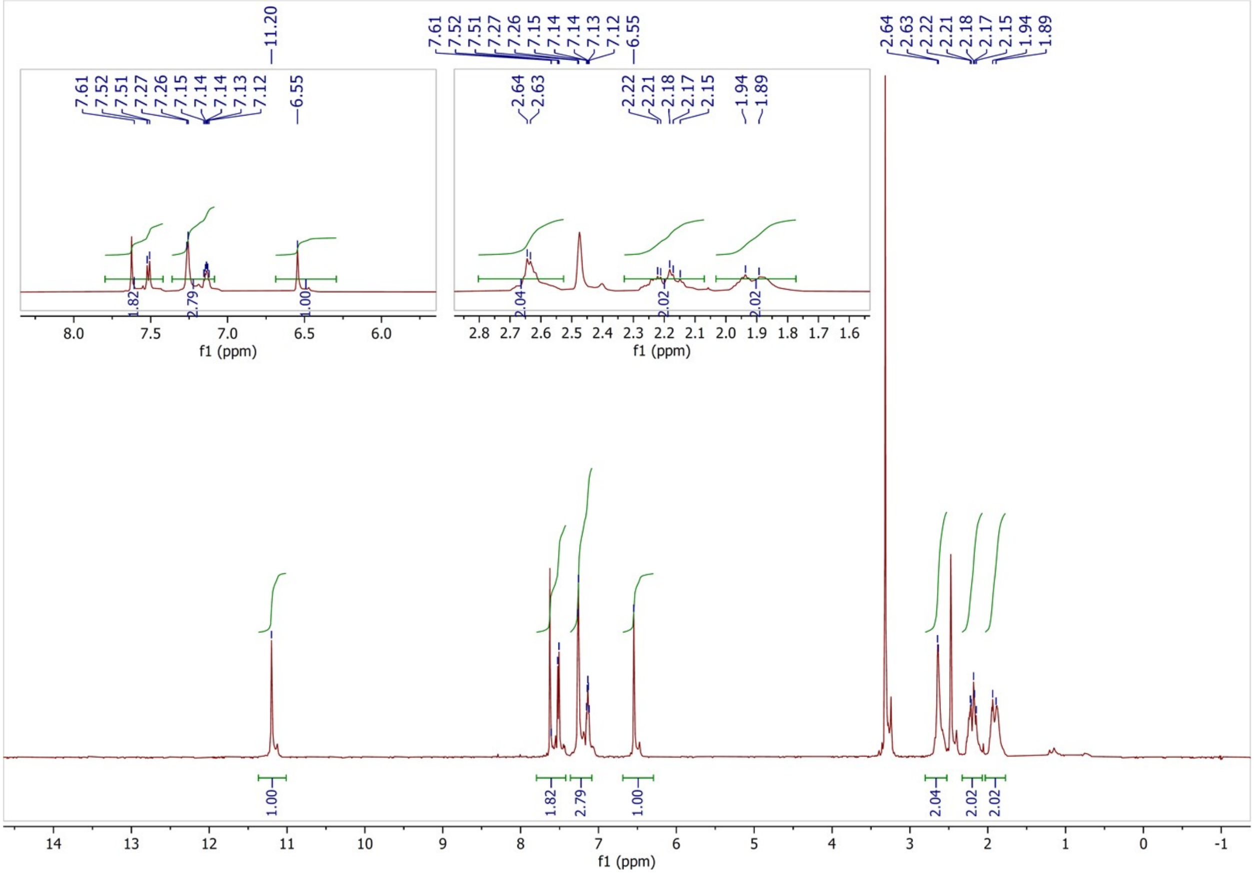
11.20
7.61
7.52
7.51
7.27
7.26
7.15
7.14
7.14
7.13
7.12
6.55
2.64
2.63
2.22
2.21
2.18
2.17
2.15
1.94
1.89
1.00
1.82
2.79
1.00
2.04
2.02
2.02
f1 (ppm)

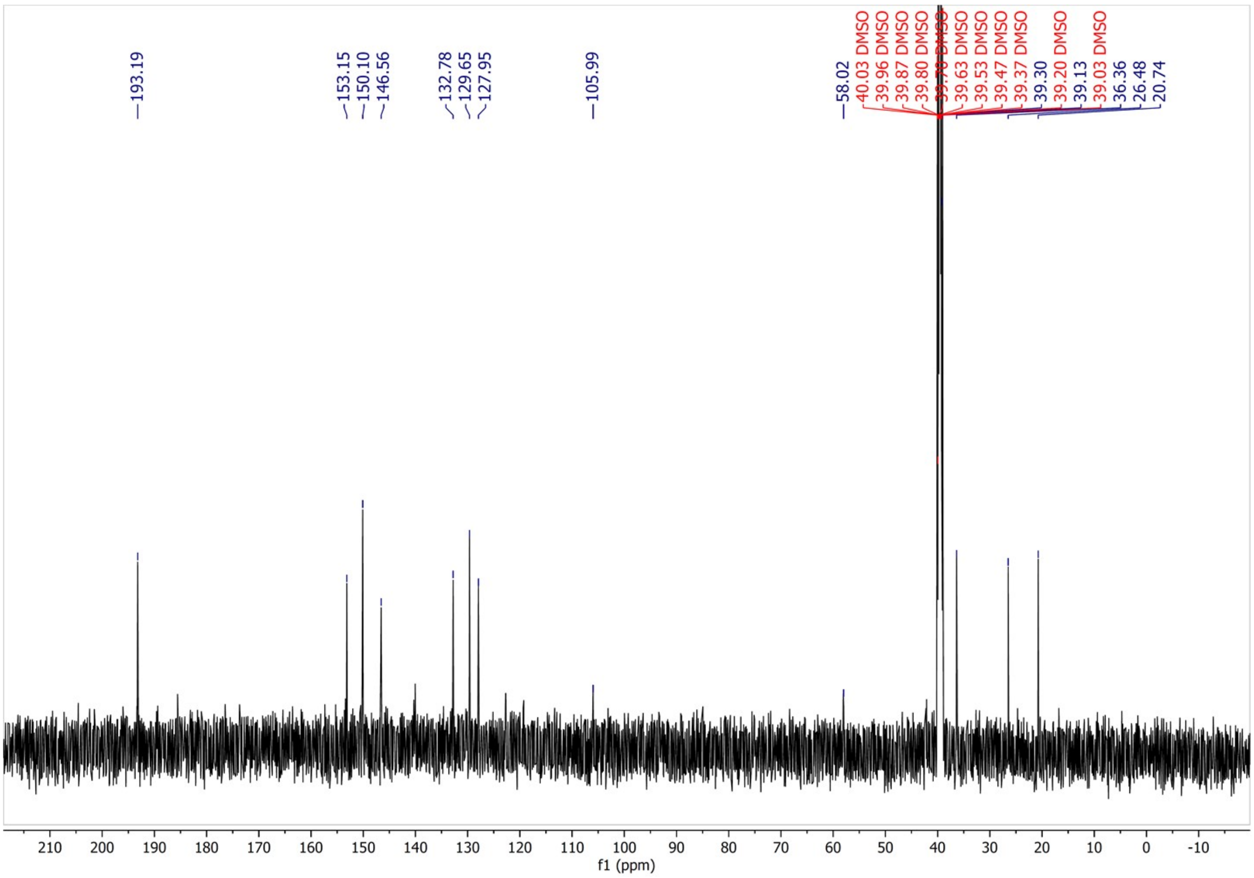
193.19
153.15
150.10
146.56
132.78
129.65
127.95
105.99
58.02
40.03 DMSO
39.96 DMSO
39.87 DMSO
39.80 DMSO
39.63 DMSO
39.53 DMSO
39.47 DMSO
39.37 DMSO
39.30
39.20 DMSO
39.13
39.03 DMSO
36.36
26.48
20.74
f1 (ppm)

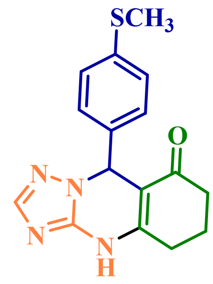
SCH3
O
N
N
N
N
H

22a

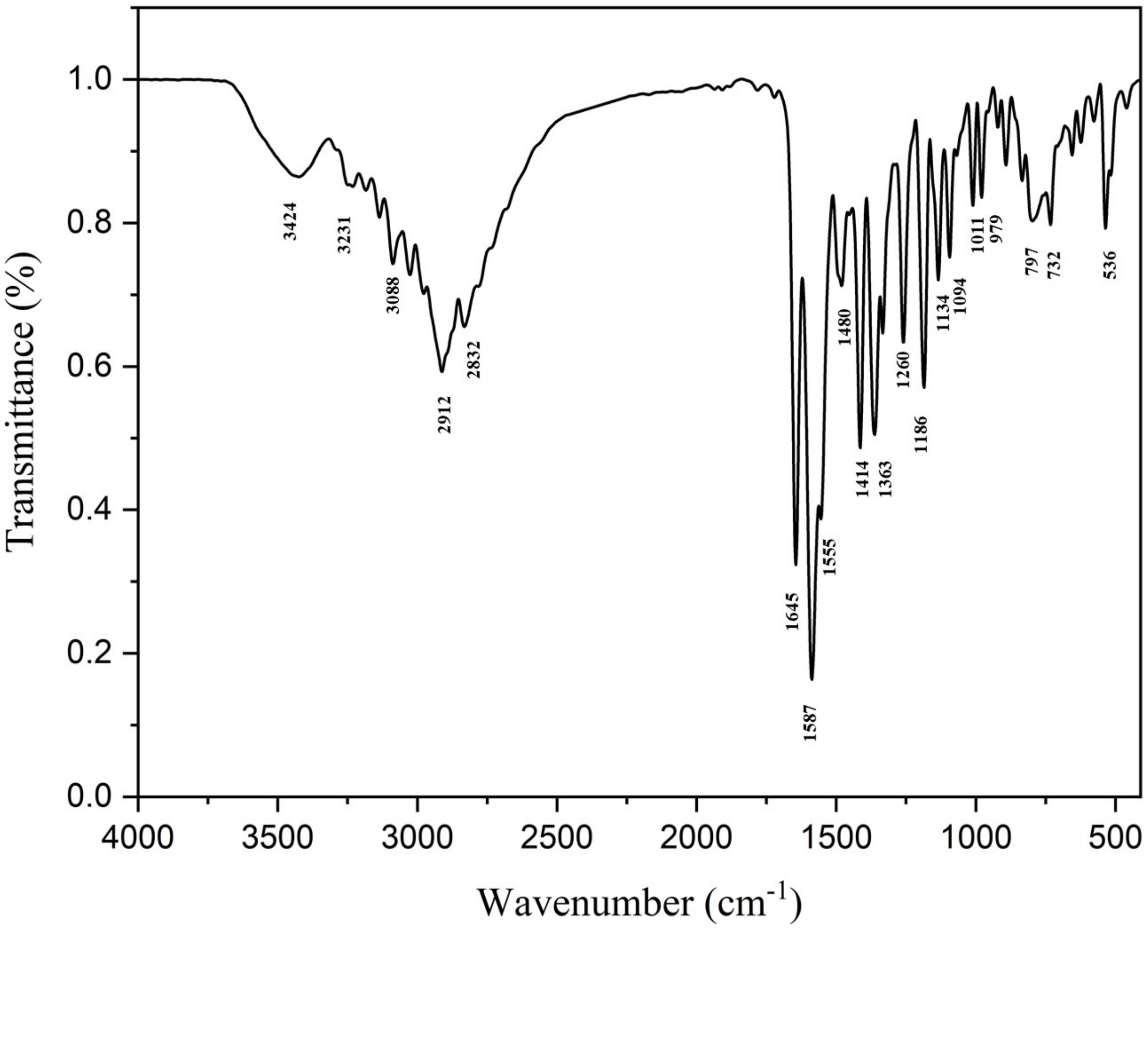
Transmittance (%)
Wavenumber (cm-1)
1.0
0.8
0.6
0.4
0.2
0.0
4000
3500
3000
2500
2000
1500
1000
500
3424
3231
3088
2912
2832
1645
1587
1555
1480
1414
1363
1260
1186
1134
1094
1011
979
797
732
536

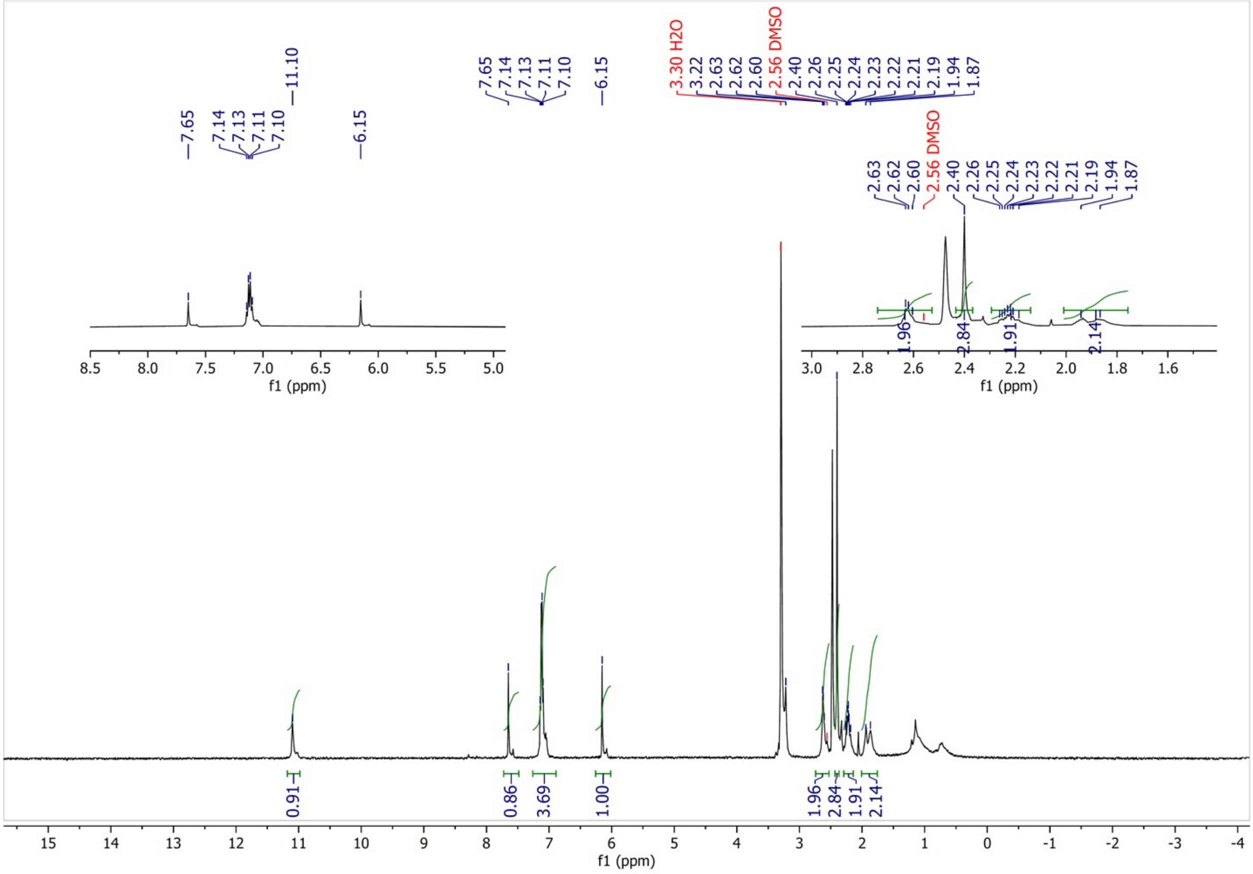

3.30 H2O
2.56 DMSO
11.10
7.65
7.14
7.13
7.11
7.10
6.15
3.22
2.63
2.62
2.60
2.40
2.26
2.25
2.24
2.23
2.22
2.21
2.19
1.94
1.87
0.91
0.86
3.69
1.00
1.96
2.84
1.91
2.14
f1 (ppm)

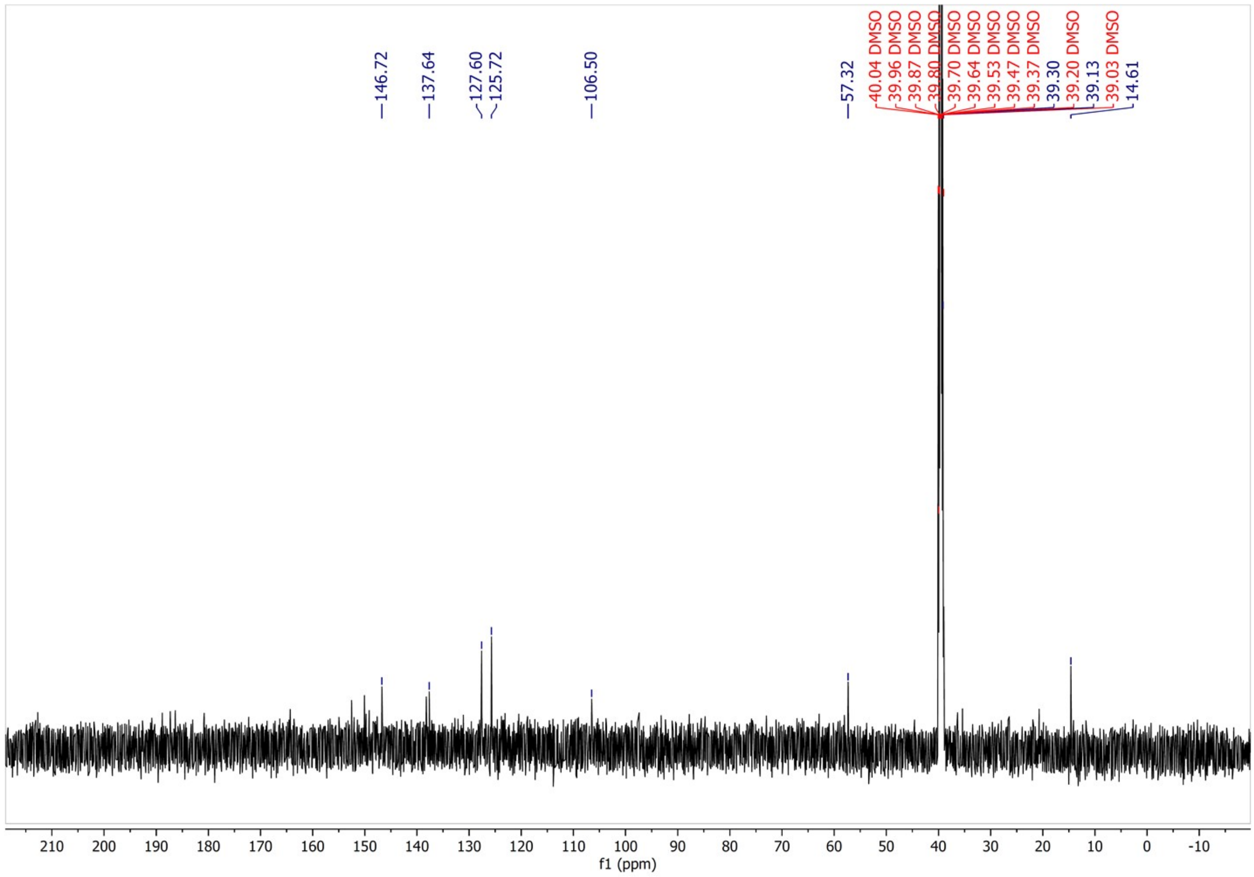
146.72
137.64
127.60
125.72
106.50
57.32
40.04 DMSO
39.96 DMSO
39.87 DMSO
39.80 DMSO
39.70 DMSO
39.64 DMSO
39.53 DMSO
39.47 DMSO
39.37 DMSO
39.30
39.20 DMSO
39.13
39.03 DMSO
14.61
210 200 190 180 170 160 150 140 130 120 110 100 90 80 70 60 50 40 30 20 10 0 -10
f1 (ppm)

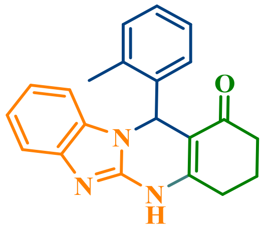
O
N
N
N
H

11b

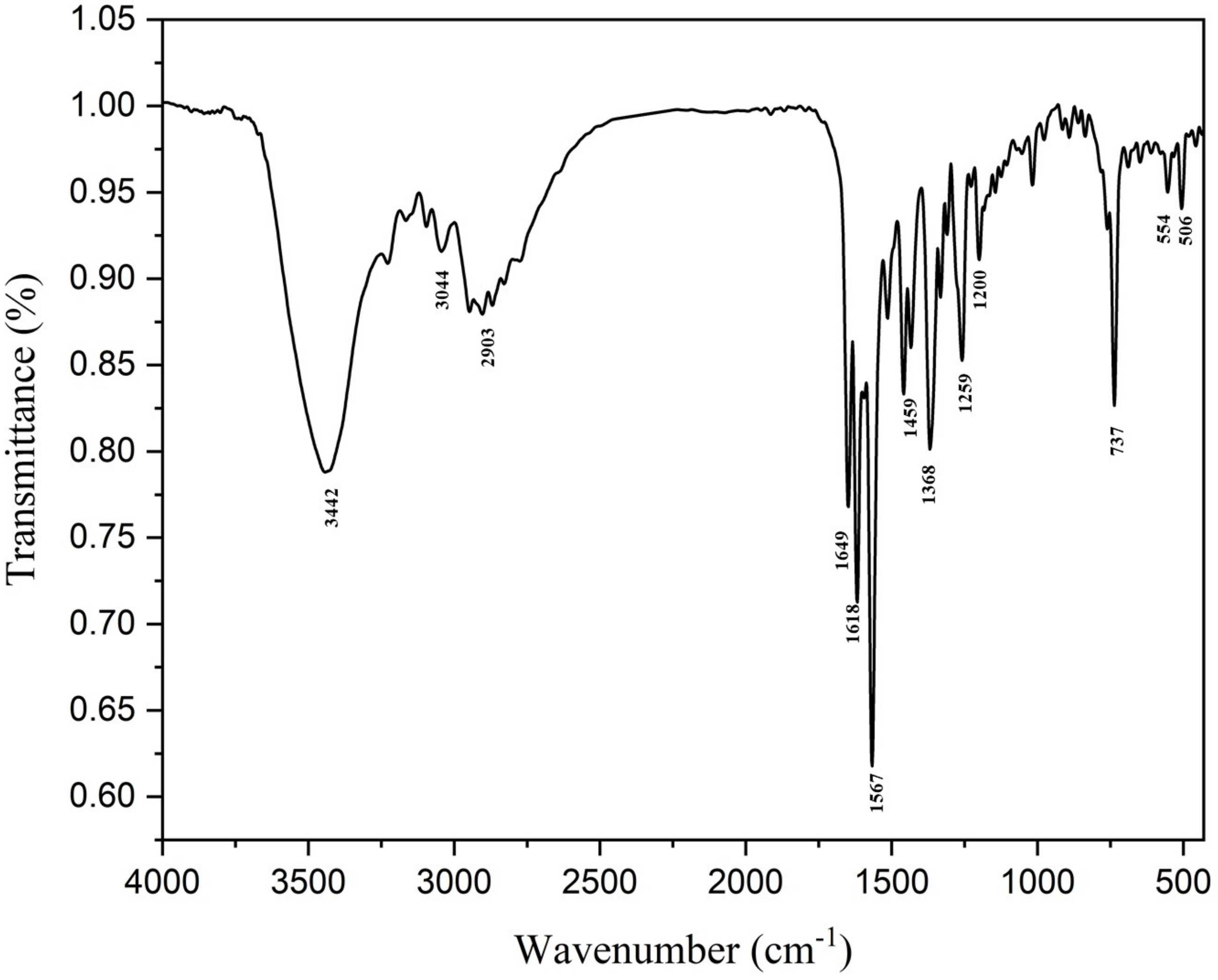
Transmittance (%)
Wavenumber (cm-1)
1.05
1.00
0.95
0.90
0.85
0.80
0.75
0.70
0.65
0.60
4000
3500
3000
2500
2000
1500
1000
500
3442
3044
2903
1649
1618
1567
1459
1368
1259
1200
737
554
506

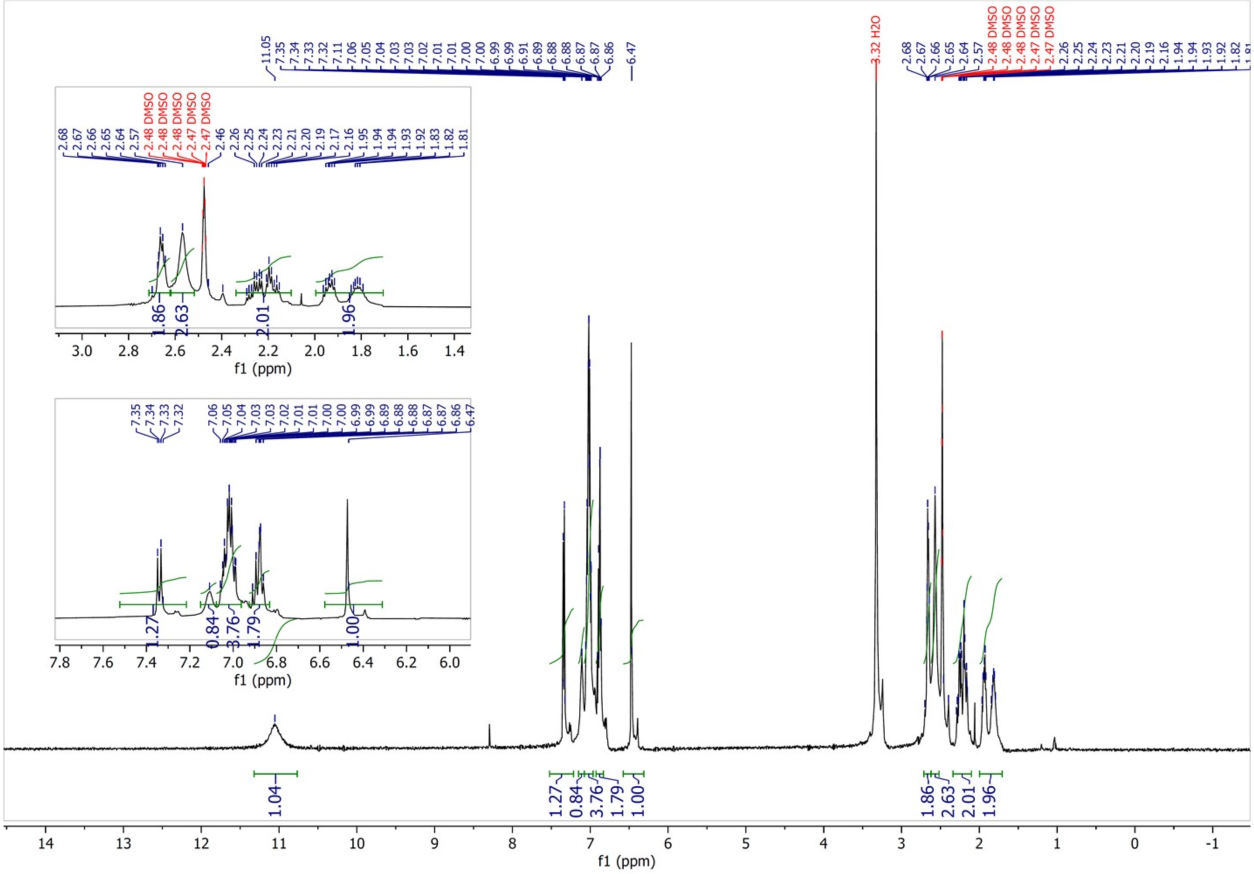

11.05
3.32 H2O
6.47
1.04
1.27
0.84
3.76
1.79
1.00
1.86
2.63
2.01
1.96
f1 (ppm)

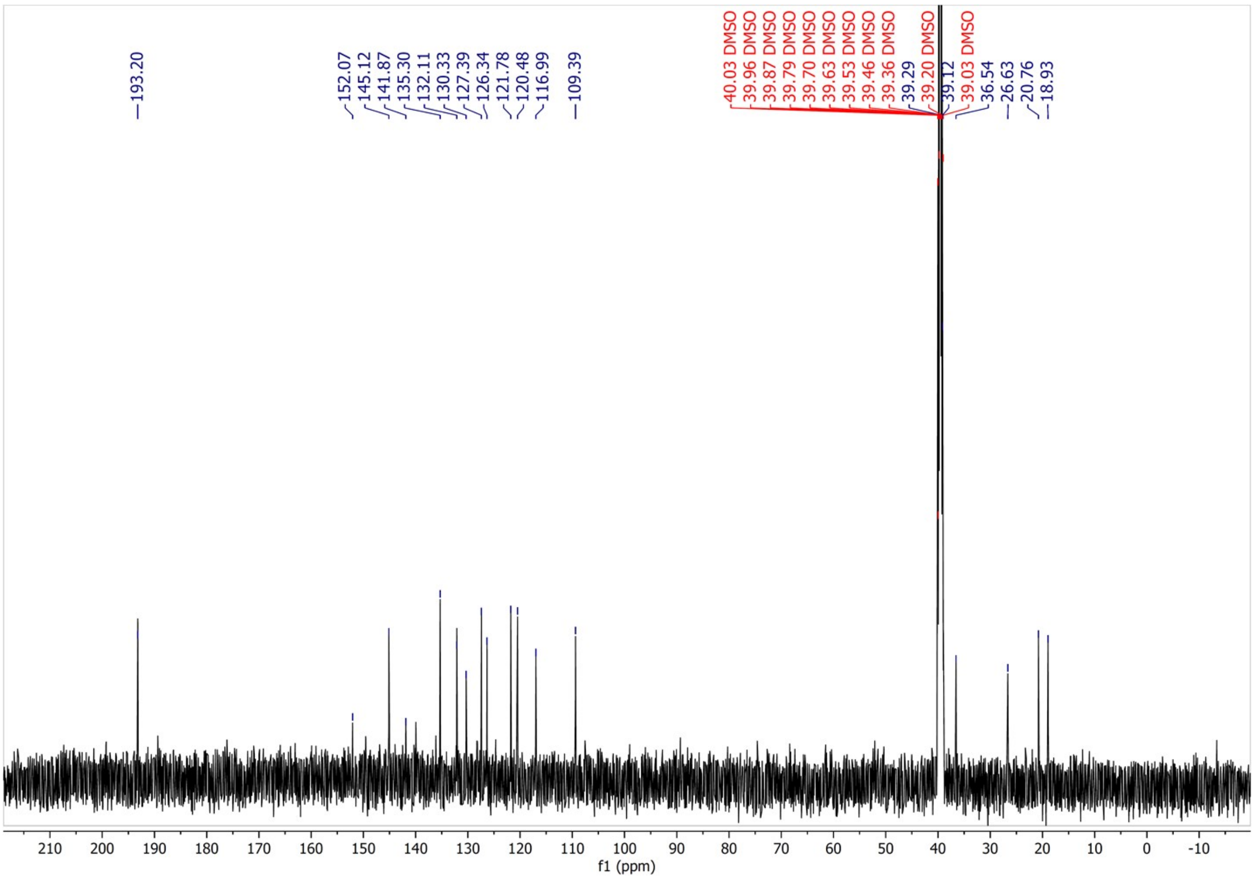

193.20
152.07
145.12
141.87
135.30
132.11
130.33
127.39
126.34
121.78
120.48
116.99
109.39
40.03 DMSO
39.96 DMSO
39.87 DMSO
39.79 DMSO
39.70 DMSO
39.63 DMSO
39.53 DMSO
39.46 DMSO
39.36 DMSO
39.29
39.20 DMSO
39.12
39.03 DMSO
36.54
26.63
20.76
18.93
f1 (ppm)

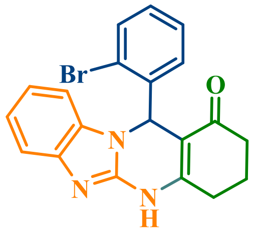

13b

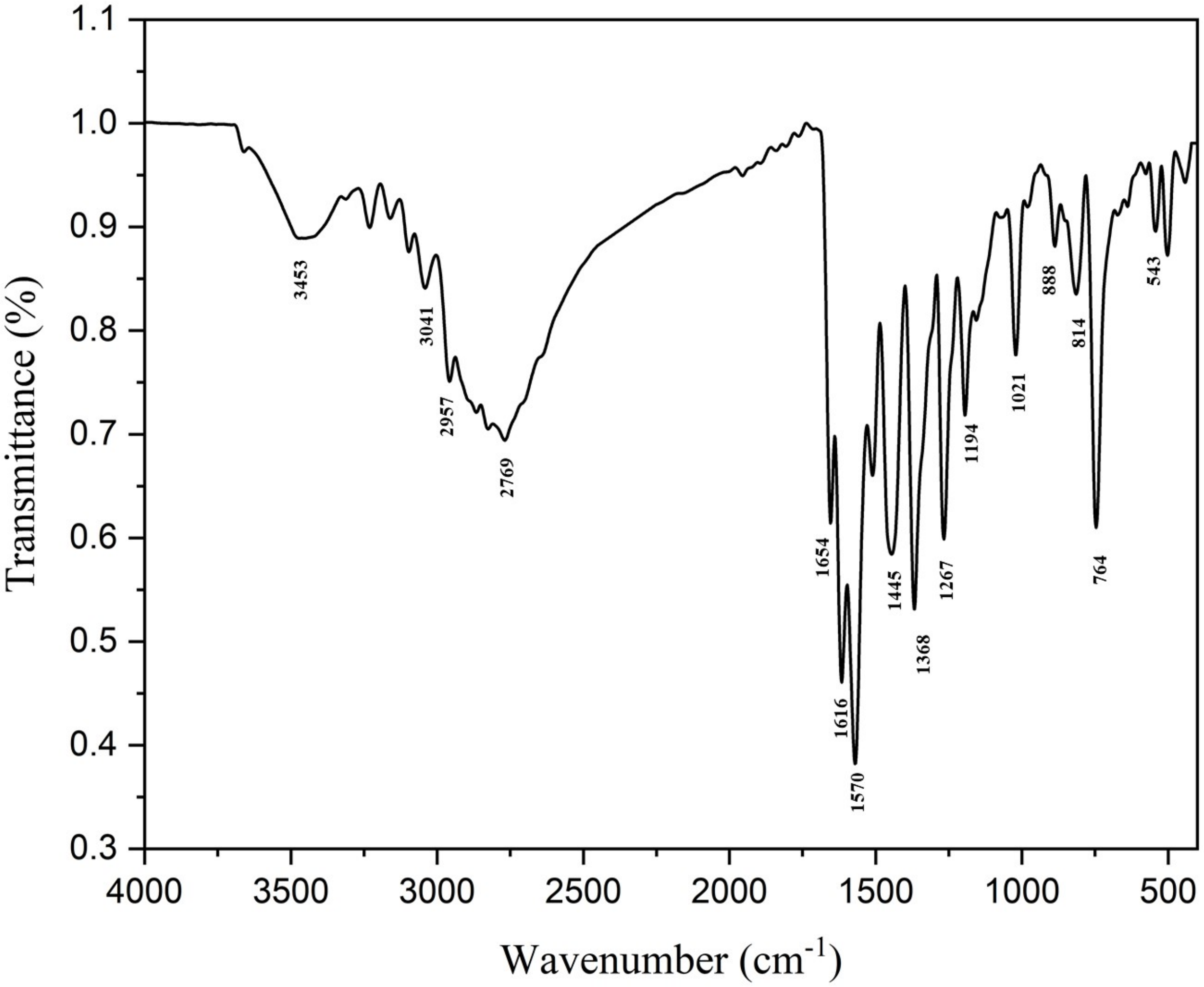
1.1
1.0
0.9
0.8
0.7
0.6
0.5
0.4
0.3
Transmittance (%)
4000
3500
3000
2500
2000
1500
1000
500
Wavenumber (cm-1)
3453
3041
2957
2769
1654
1616
1570
1445
1368
1267
1194
1021
888
814
764
543

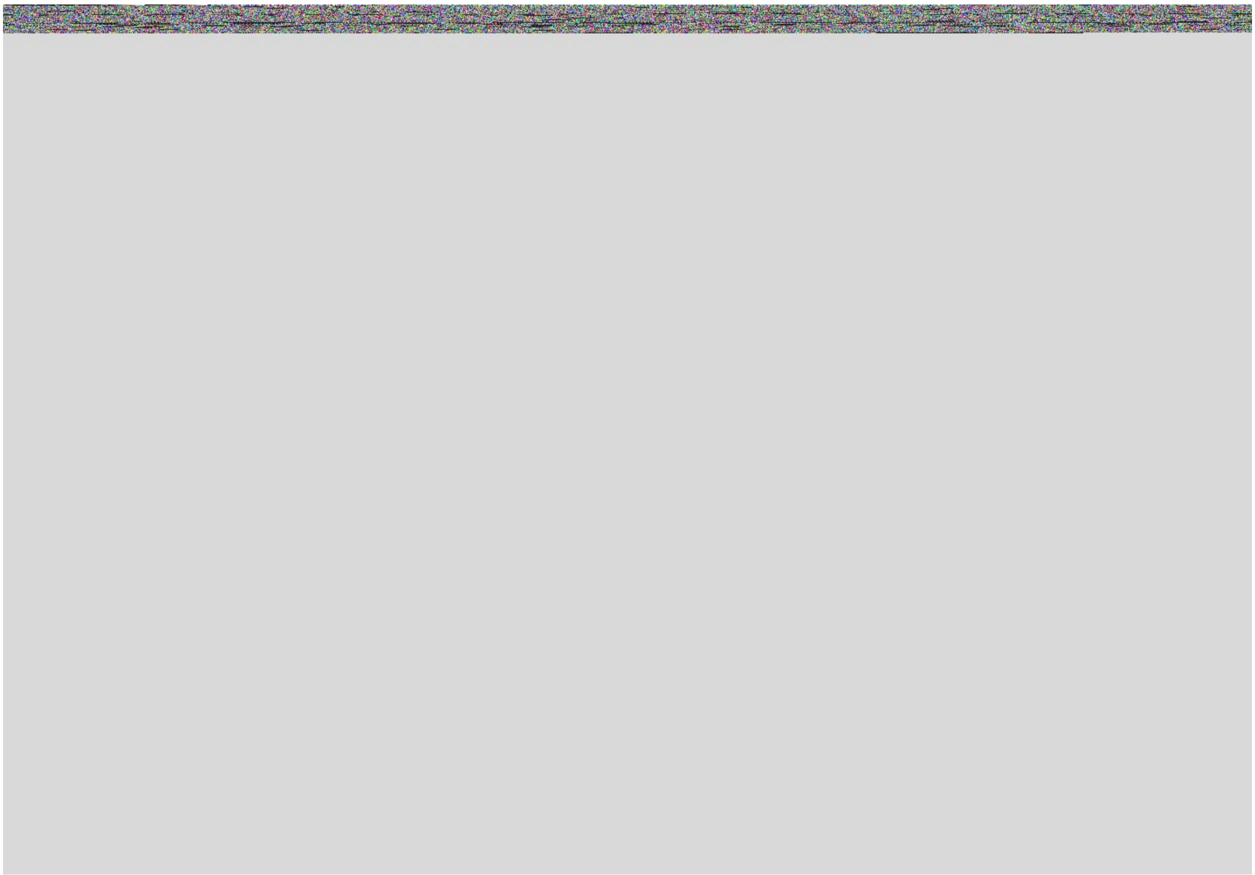

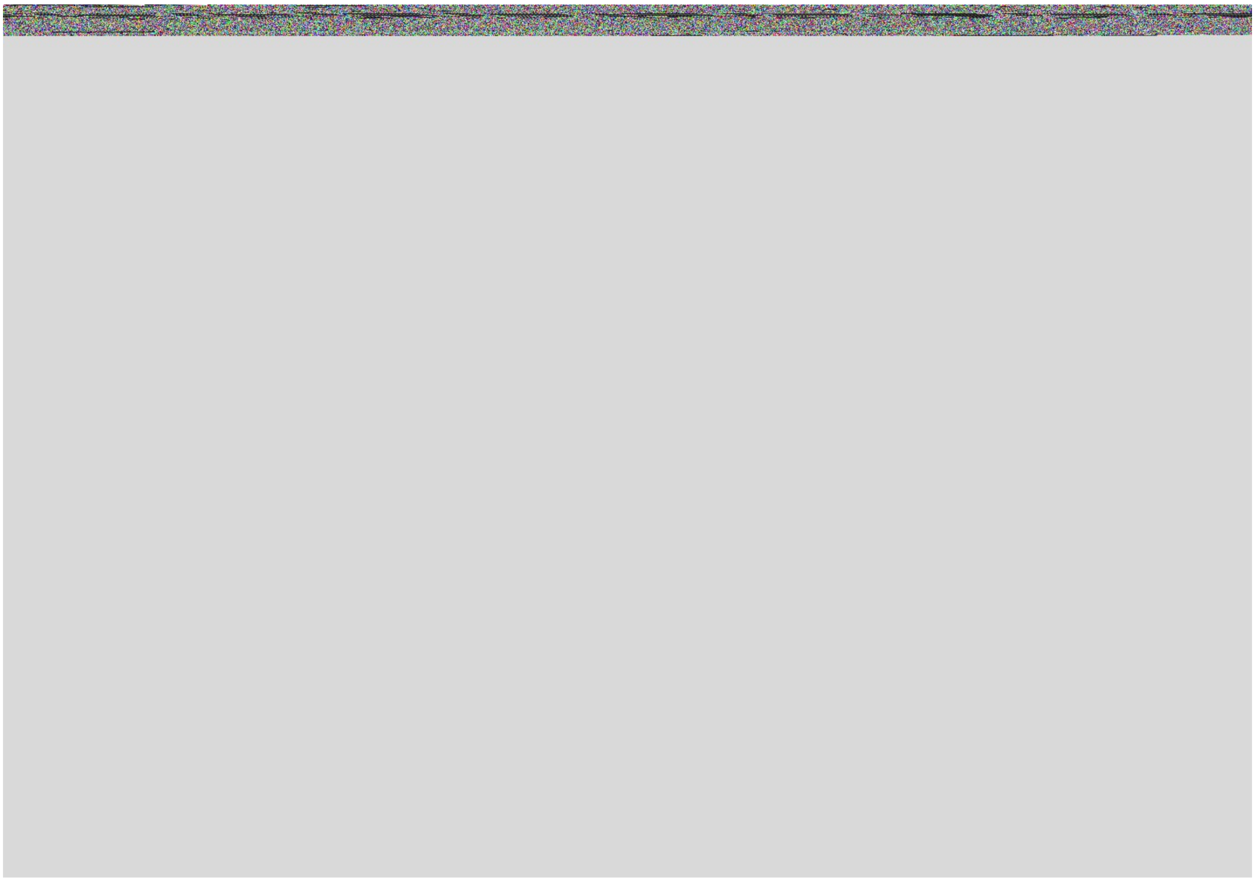

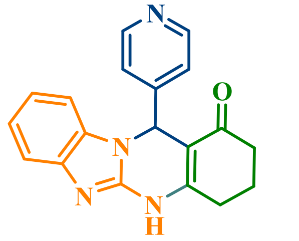

15b

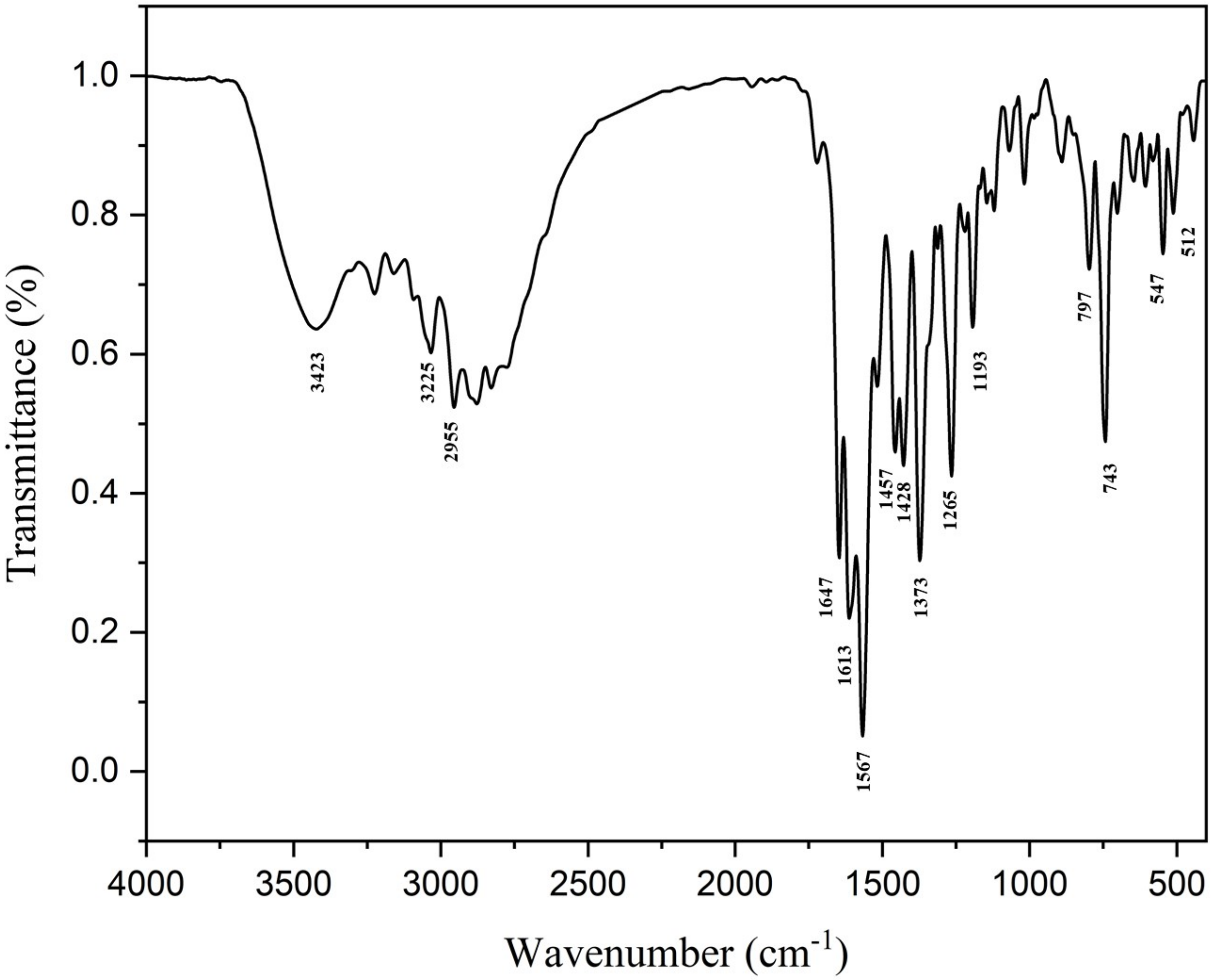
Transmittance (%)
Wavenumber (cm-1)
1.0
0.8
0.6
0.4
0.2
0.0
4000
3500
3000
2500
2000
1500
1000
500
3423
3225
2955
1647
1613
1567
1457
1428
1373
1265
1193
797
743
547
512

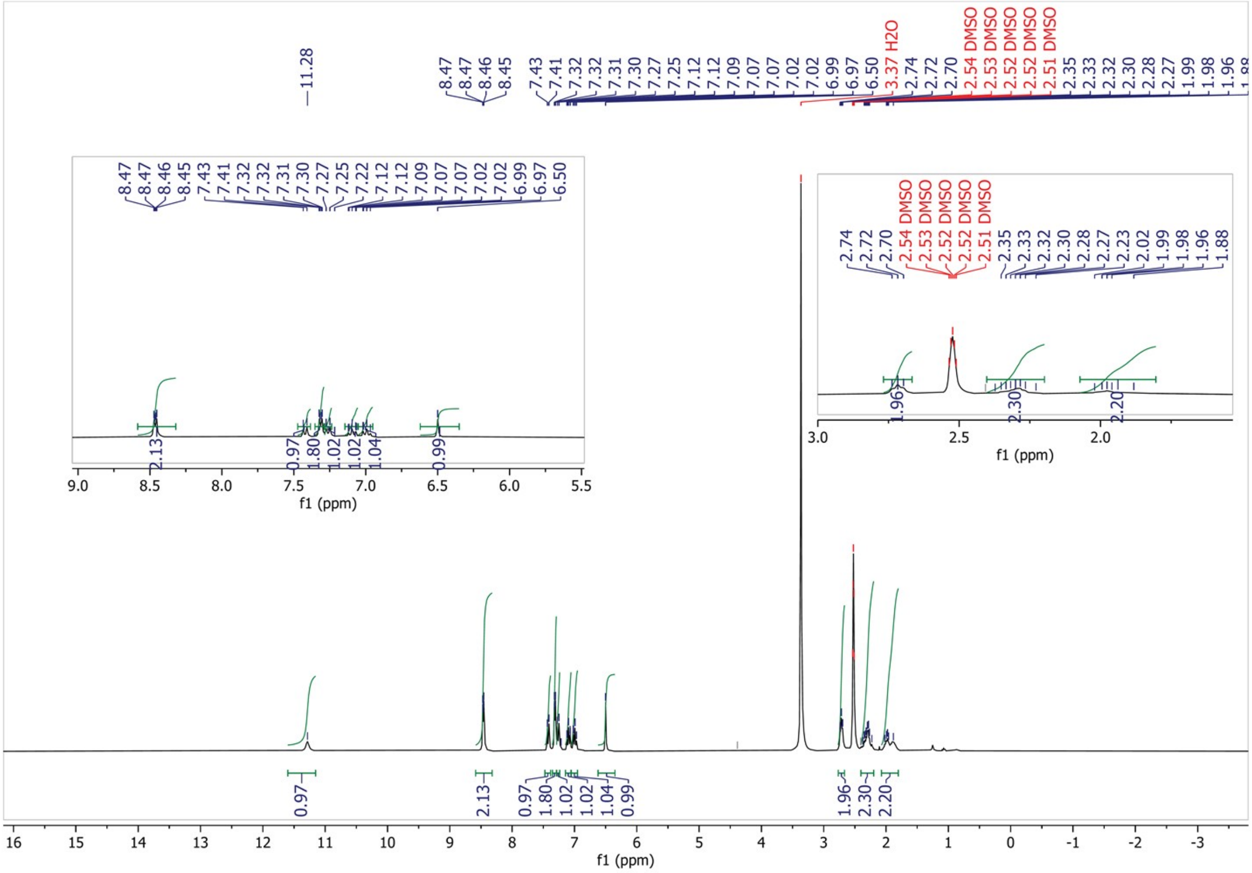
11.28
8.47 8.47 8.46 8.45 7.43 7.41 7.32 7.32 7.31 7.30 7.27 7.25 7.12 7.12 7.09 7.07 7.07 7.02 7.02 6.99 6.97 6.50
3.37 H2O
2.74 2.72 2.70
2.54 DMSO 2.53 DMSO 2.52 DMSO 2.52 DMSO 2.51 DMSO
2.35 2.33 2.32 2.30 2.28 2.27 1.99 1.98 1.96
8.47 8.47 8.46 8.45 7.43 7.41 7.32 7.32 7.31 7.30 7.27 7.25 7.22 7.12 7.12 7.09 7.07 7.07 7.02 7.02 6.99 6.97 6.50
2.13 0.97 1.80 1.02 1.02 1.04 0.99
9.0 8.5 8.0 7.5 7.0 6.5 6.0 5.5
f1 (ppm)
2.74 2.72 2.70 2.54 DMSO 2.53 DMSO 2.52 DMSO 2.52 DMSO 2.51 DMSO 2.35 2.33 2.32 2.30 2.28 2.27 2.23 2.02 1.99 1.98 1.96 1.88
1.96 2.30 2.20
3.0 2.5 2.0
f1 (ppm)
0.97 2.13 0.97 1.80 1.02 1.02 1.04 0.99 1.96 2.30 2.20
16 15 14 13 12 11 10 9 8 7 6 5 4 3 2 1 0 -1 -2 -3
f1 (ppm)

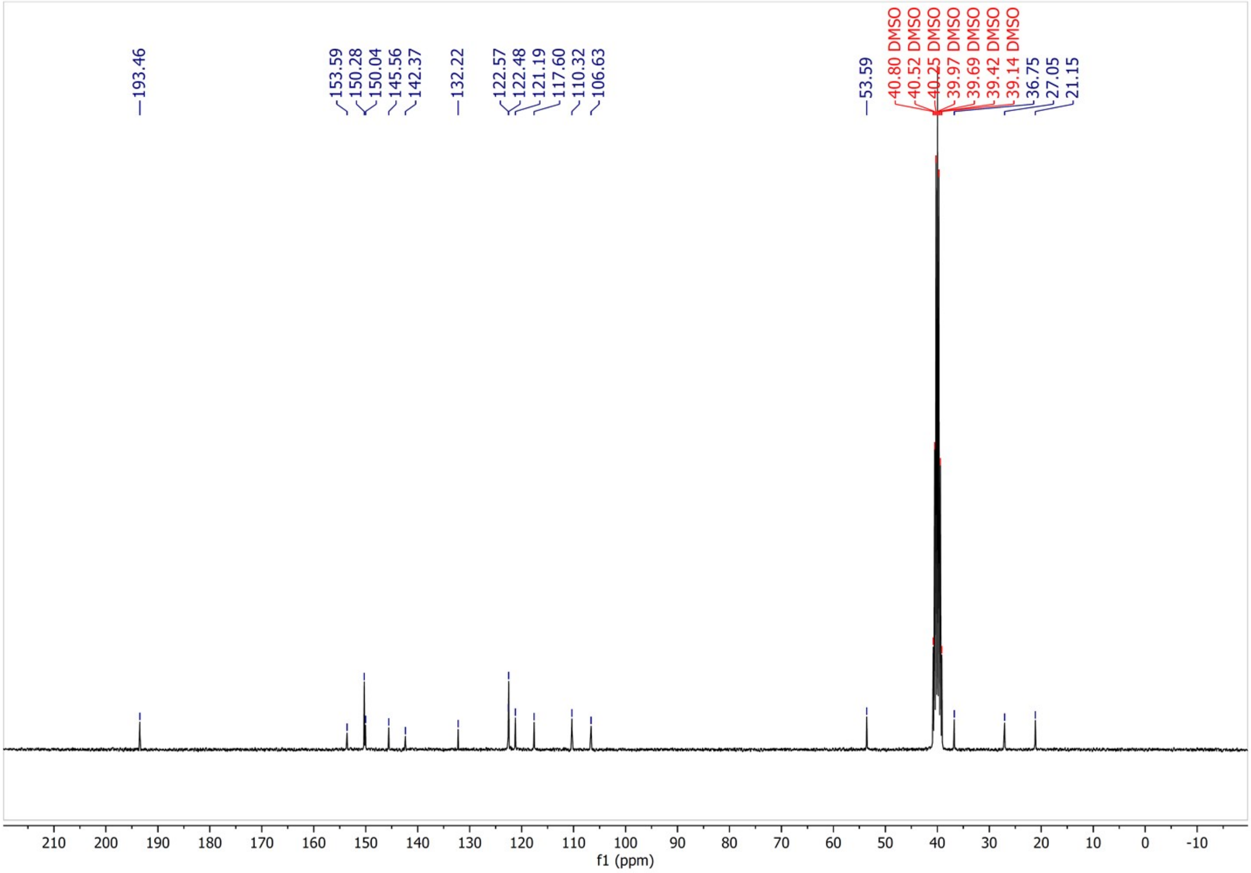
193.46
153.59 150.28 150.04 145.56 142.37
132.22
122.57 122.48 121.19 117.60 110.32 106.63
53.59
40.80 DMSO 40.52 DMSO 40.25 DMSO 39.97 DMSO 39.69 DMSO 39.42 DMSO 39.14 DMSO
36.75 27.05 21.15
210 200 190 180 170 160 150 140 130 120 110 100 90 80 70 60 50 40 30 20 10 0 -10
f1 (ppm)
